# circEPB41L2 blocks the progression and metastasis in non-small cell lung cancer by promoting TRIP12-triggered PTBP1 ubiquitylation

**DOI:** 10.1038/s41420-024-01836-4

**Published:** 2024-02-10

**Authors:** Yan Wang, Yihao Wang, Chunjie Wu, Yunfei Ji, Pingfu Hou, Xueqing Wu, Zhongwei Li, Minle Li, Sufang Chu, Qianqian Ning, Bo Xu, Junnian Zheng, Jin Bai

**Affiliations:** 1 Cancer Institute, Xuzhou Medical University, Xuzhou, Jiangsu China; 2 Jiangsu Center for the Collaboration and Innovation of Cancer Biotherapy, Cancer Institute, Xuzhou Medical University, Xuzhou, Jiangsu China; 3 Center of Clinical Oncology, Affiliated Hospital of Xuzhou Medical University, Xuzhou, Jiangsu China; 4 Department of Pharmacy, Affiliated Hospital of Xuzhou Medical University, Xuzhou, Jiangsu China; 5 Jiangsu Key Laboratory of New Drug Research and Clinical Pharmacy of Xuzhou Medical University, Xuzhou, Jiangsu China

**Keywords:** Non-small-cell lung cancer, Metastasis

## Abstract

The metastasis of non-small cell lung cancer (NSCLC) is the leading death cause of NSCLC patients, which requires new biomarkers for precise diagnosis and treatment. Circular RNAs (circRNAs), the novel noncoding RNA, participate in the progression of various cancers as microRNA or protein sponges. We revealed the mechanism by which circEPB41L2 (hsa_circ_0077837) blocks the aerobic glycolysis, progression and metastasis of NSCLC through modulating protein metabolism of PTBP1 by the E3 ubiquitin ligase TRIP12. With ribosomal RNA-depleted RNA seq, 57 upregulated and 327 downregulated circRNAs were identified in LUAD tissues. circEPB41L2 was selected due to its dramatically reduced levels in NSCLC tissues and NSCLC cells. Interestingly, circEPB41L2 blocked glucose uptake, lactate production, NSCLC cell proliferation, migration and invasion in vitro and in vivo. Mechanistically, acting as a scaffold, circEPB41L2 bound to the RRM1 domain of the PTBP1 and the E3 ubiquitin ligase TRIP12 to promote TRIP12-mediated PTBP1 polyubiquitylation and degradation, which could be reversed by the HECT domain mutation of TRIP12 and circEPB41L2 depletion. As a result, circEPB41L2-induced PTBP1 inhibition led to PTBP1-induced PKM2 and Vimentin activation but PKM1 and E-cadherin inactivation. These findings highlight the circEPB41L2-dependent mechanism that modulates the “Warburg Effect” and EMT to inhibit NSCLC development and metastasis, offering an inhibitory target for NSCLC treatment.

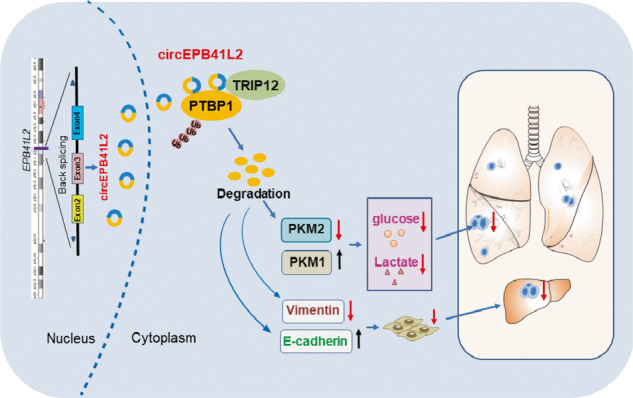

## Introduction

Lung cancer (LC) is the leading cause of cancer-related death worldwide [1]. Non-small cell lung cancer (NSCLC) which contains lung adenocarcinoma (LUAD), lung squamous cell carcinoma (LUSC) and large cell carcinoma accounts for ~85% of all LC cases [2, 3]. The increasing morbidity and mortality rates of LC require precise biomarkers for early diagnosis and treatment [3, 4].

Circular RNAs (circRNAs), characterised by covalently closed continuous loops structures without 5’ and 3’ ends, are the products of back splicing events or noncolinear splicing reactions of precursor mRNAs [5–8]. The unique circular structure of circRNAs ensures their higher stability than their linear counterparts in tissues and plasma [6, 9–11]. The reported canonical function of these circular molecules is to adsorb microRNAs (miRNAs) or proteins as sponges to participate in the tumorigenesis and progression of various cancers [12–15]. Intriguingly, circRNAs perform noncanonical functions that control the fate of proteins by acting as aits, scaffolds, chaperone molecules or translation templates and regulate the development and metastasis of various cancers, including lung cancer [16–21]. For example, circCTNNB1 drives tumorigenesis and aggressiveness by binding to DDX3 to facilitate its interaction with Yin Yang 1 and transactivate β-catenin [18].

Numerous RNA binding proteins, termed RBPs, are reportedly responsible for the biogenesis of circRNAs as trans-acting factors [22–24]. Unexpectedly, certain circRNAs were observed to perform novel functions to stabilise or decrease the expression of RBPs including human antigen R (HuR) and polypyrimidine tract-binding protein 1 (PTBP1) which are involved in tumour progression [19, 25, 26]. circRHOBTB3 (hsa_circ_0007444) directly binds to HuR to accelerate its ubiquitination and degradation induced by the E3 ubiquitin ligase β-Trcp1, limiting the mRNA expression of PTBP1, a target of HuR, and thereby abolishing PTBP1-induced CRC metastasis [19]. PTBP1, a multifunctional RNA-binding protein, is involved in metabolic reprogramming, cell motility and the cell cycle [27, 28]. Aerobic glycolysis is the most prominent character of energy metabolism remodelling in tumour development [27]. Our previous study revealed that PTBP1 promoted the progression of renal cell carcinoma by stimulating the hypoxia inducible factor-1α pathway [28]. However, the mechanism of circRNA-mediated PTBP1 regulation in aerobic glycolysis, development and metastasis of NSCLC remains unknown.

Utilising ribosomal RNA-depleted RNA sequencing (RNA-seq), we identified that circEPB41L2 downregulation in primary and advanced NSCLC tissues were relevant to unfavourable outcomes in NSCLC patients. Furthermore, circEPB41L2 inhibited glucose uptake and lactate production, progression and metastasis in NSCLC by binding to the RRM1 domain of the PTBP1 protein to promote its interaction with the E3 ligase TRIP12, which induced PTBP1 polyubiquitylation and degradation and blocked PTBP1-induced PKM2 and Vimentin accumulation. These findings highlight a circRNA-dependent mechanism that modulates PTBP1 metabolism via interacting with the ubiquitin ligase to repress aerobic glycolysis, progression and metastasis in NSCLC. Therefore, circEPB41L2 may act as a suppressor of NSCLC.

## Results

### Profiling of differentially expressed circRNAs in LUAD tissues

**Fig. 1:**
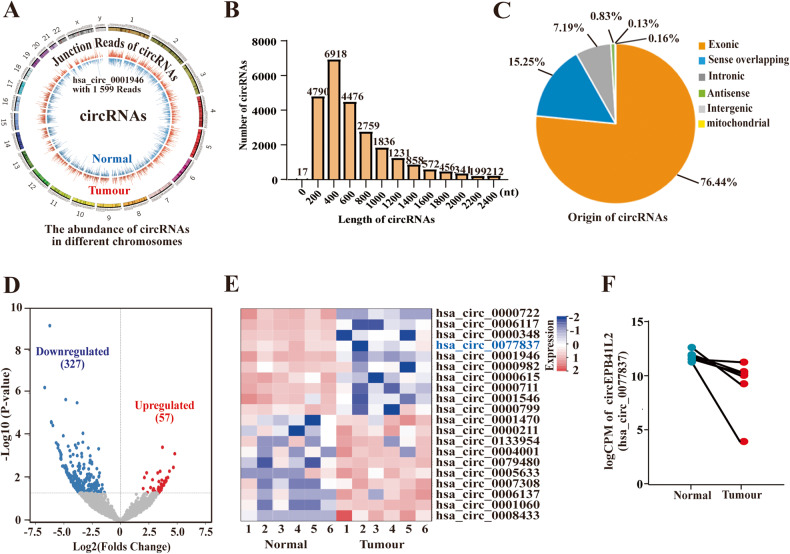
Profiling of differentially expressed circRNAs in LUAD tissues. **A** The back-spliced Reads distribution of screened circRNAs in tumour and adjacent normal tissues from different chromosomes of RNA-seq. **B** The length distribution of identified circRNAs. *x*-axis: the length of detected circRNAs. *y*-axis: the numbers of detected circRNAs. **C** The origin and the numbers of identified circRNAs derived from different genomic regions. **D** The differentially expressed circRNAs in LUAD and adjacent normal tissues. **E** Hierarchical clustering heatmap indicated the top ten downregulated and upregulated circRNAs in LUAD and adjacent normal tissues. **F** The expression of circEPB41L2(hsa_circ_0077837) in LUAD and adjacent normal tissues by RNA-seq.

The clean reads and reads per million mapped reads (RPM) were used to measure the abundance of circRNAs. The definition of normalised circRNA was supported by at least two unique back-spliced reads in one sample as previously reported [20, 29]. RNA-seq results indicated that the number of junction reads of circRNAs from different chromosomes in tumour tissues and normal tissues ranged from 2–1599. The reads in tumour tissues were mostly less than 50 compared to normal controls, implying a lower abundance of circRNAs in tumour tissues (Fig. 1A). The majority of identified circRNAs were less than 1600 nucleotides (Fig. 1B). Moreover, the origin of the identified circRNAs included the nuclear and mitochondrial genome (Fig. 1C). The data of exon circRNAs (76.44%), sense overlapping circRNAs (15.25%), intronic circRNAs (7.19%), antisense circRNAs (0.83%), intergenic circRNAs (0.13%) from the nuclear genome and mitochondrial genome-generated circRNAs (0.16%) suggested the diversity in circRNA origin.

Notably, 327 downregulated and 57 upregulated circRNAs were identified in tumour tissues according to the criterion [(FDR < 0.01, |logFC | ≥ 2)] as shown in Fig. 1D. The top ten downregulated and upregulated circRNAs were listed in Fig. 1E. The reads and expression of hsa_circ_0077837 (circEPB41L2) were dramatically reduced in tumour tissues compared to paired controls (Fig. 1E, F), and circEPB41L2 was screened for subsequent study.

### Characteristics of circEPB41L2 in NSCLC cells and tissues

**Fig. 2:**
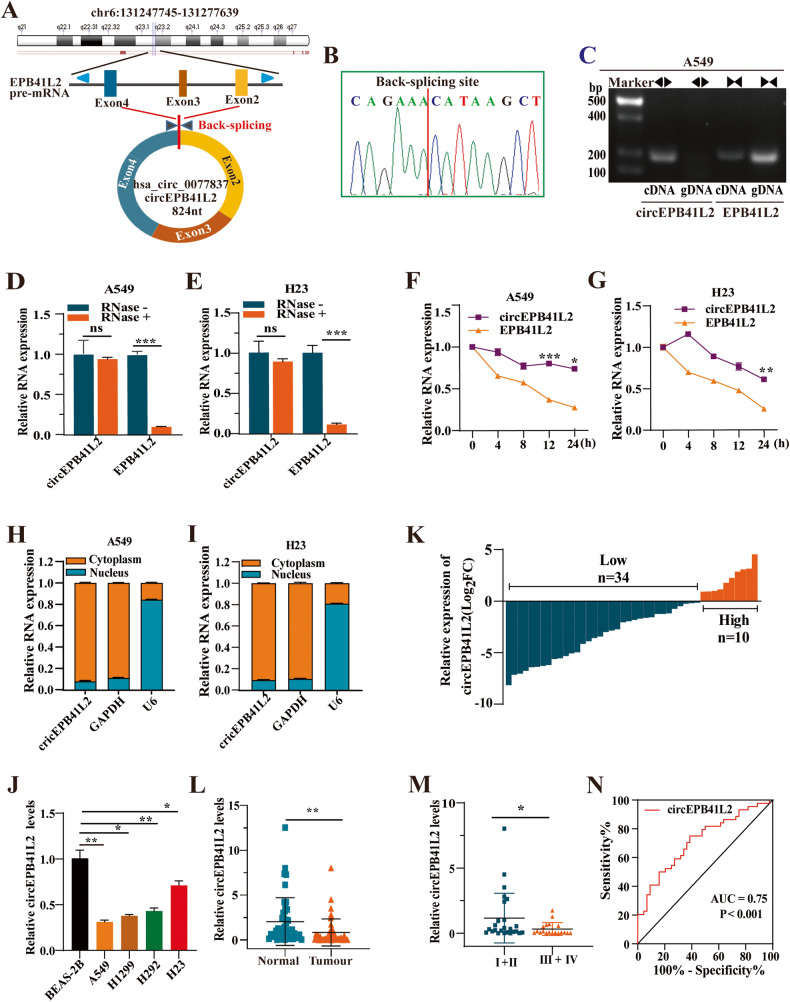
Characteristics of circEPB41L2 in NSCLC cells and tissues. **A** Illustration of the back splicing mode and length of circEPB41L2 which is generated from exons 2–4 of pre-mRNA of *EPB41L2* gene from chromosomal region. **B** Sanger sequencing of circEPB41L2 determined the back-splice junction. **C** Divergent primer (◄►) identified circEPB41L2 from cDNA but not gDNA. **D**, **E** Relative RNA levels of circEPB41L2 and *EPB41L2* mRNA treated with RNase R in A549 and H23 cells. **F**, **G** Relative RNA levels of circEPB41L2 and *EPB41L2* mRNA in different time points in A549 and H23 cells treated with Actinomycin D. **H**, **I** Relative cytoplasmic and nuclear circEPB41L2 levels in A549 and H23 cells (normalised to *GAPDH* RNA in cytoplasm and to *U6* RNA in nucleus). **J** The expression of circEPB41L2 in different NSCLC cells and human bronchial epithelial cell line (BEAS-2B). **K** The expression of circEPB41L2 in tissues of 44 NSCLC patients. **L**, **M** The expression of circEPB41L2 in the tissues of earlier (I + II) stage and advanced (III + IV) stage of NSCLC as well as adjacent controls. **N** ROC analysis indicated the potential of circEPB41L2 as a biomarker of NSCLC. Each experiment was independently conducted at least three times. The results of one representative experiment are illustrated. Data were indicated as mean ± S.D. *p* value was determined by a two-tailed paired Student’s *t* test. *p < 0.05, **p < 0.01 and ***p < 0.001 versus controls, ns no significant difference.

circEPB41L2 with a length of 824 nt is back-spliced from exons 2-4 of *EPB41L2* (Fig. 2A). Its head-to-tail splicing was confirmed by Sanger sequencing (Fig. 2B). circEPB41L2 was only amplified from complementary DNA (cDNA) by divergent primers and convergent primers (Fig. 2C). Furthermore, circEPB41L2 exhibited stronger resistance to RNase R digestion (Fig. 2D, E) and prolonged half-life (Fig. 2F, G) than its linear isoform in A549 and H23 cells. RNA nucleus/cytoplasm separation assays revealed that circEPB41L2 was mainly accumulated in the cytoplasm of A549 and H23 cells (Fig. 2H, I). circEPB41L2 levels were dramatically decreased in NSCLC cells (A549, H1299, H292 and H23 cells) compared to BEAS-2B cells (Fig. 2J). Additionally, circEPB41L2 levels were distinctly reduced in 34 tumour tissues (Fig. 2K, L) accounting for 77.27% (34/44) and in 19 advanced (III + IV tumour tissues accounting for 43.18% (Fig. 2M and Table 1). The ROC analysis results demonstrated its potential as a biomarker of NSCLC (AUC = 0.75, Fig. 2N). These data confirm the presence of endogenous circEPB41L2, and its downregulation may be involved in progression and metastasis of NSCLC as a negative marker.

**Table 1:** circEPB41L2 expression in 44 NSCLC patients.

Characteristics	Total (%)	Low (%)	High (%)	*p*
Total	44 (100.00)	34 (77.27)	10 (22.73)	
Gender				
Male	27 (61.36)	20 (74.07)	7 (25.93)	0.716
Female	17 (38.63)	14 (82.35)	3 (17.65)	
Age (years)				
<65	24 (54.55)	20 (83.33)	4 (16.67)	0.472
≥65	20 (45.45)	14 (70.00)	6 (30.00)	
TNM stage				
I + II	25 (56.82)	16 (64.00)	9 (36.00)	**0.027**
III + IV	19 (43.18)	18 (94.74)	1 (5.26)	
Tumour size(cm)				
<5	34 (77.27)	25 (73.53)	9 (26.47)	0.411
≥5	10 (22.73)	9 (90.00)	1 (10.00)	
Metastasis				
Negative	26 (59.09)	20 (76.92)	6 (23.08)	1.000
Positive	18 (40.91)	14 (77.78)	4 (22.22)	
Histology				
LUAD	30 (68.18)	23 (76.67)	7 (23.33)	1.000
LUSC	14 (31.82)	11 (78.57)	3 (21.43)	

*p* values are from *χ*^2^ test.

*LUAD* lung adenocarcinoma, *LUSC* lung squamous carcinoma.

The bold values indicate the significant difference in TNM stage.

### circEPB41L2 inhibits NSCLC progression and metastasis in vitro and in vivo

**Fig. 3:**
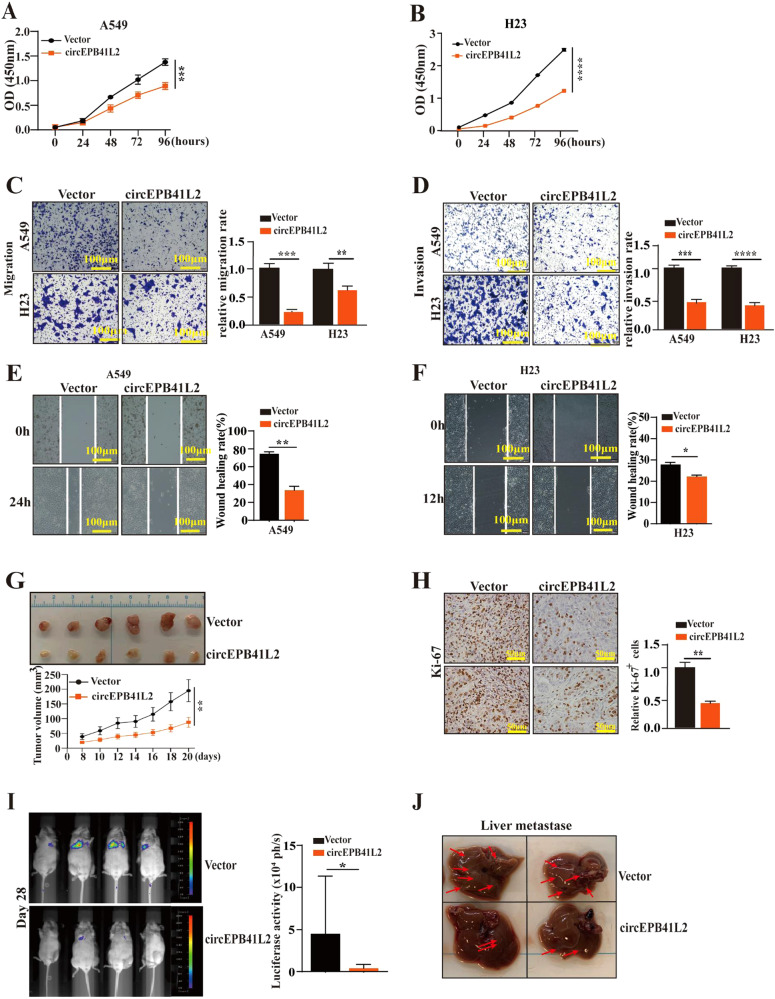
circEPB41L2 inhibits NSCLC progression and metastasis in vitro and in vivo. **A**, **B** Effects of circEPB41L2 on the cell proliferation of A549 and H23 cells measured by CCK8 assay. **C** Effects of circEPB41L2 on the cell migration ability of A549 and H23 cells detected by transwell assays. **D** Effects of circEPB41L2 on the cell invasiveness ability of A549 and H23 cells detected by transwell assays. **E**, **F** Effects of circEPB41L2 on cell migration abilities of A549 and H23 cells examined by wound-healing assays. **G**, **H** Effects of circEPB41L2 on the tumour growth of NSCLC in vivo by evaluating tumour volume and tumour growth curve and Ki-67 expression by IHC. Nude mice xenografts were formed by A549 cells stable transfected with Vector or circEPB41L2. **I** Effects of circEPB41L2 on the tumour metastasis of NSCLC in vivo. Nude mouse metastasis model was formed by A549 cells stable transfected with Vector or circEPB41L2 for 4 weeks. Bioluminescence imaging was used to examine the Firefly luciferase activity. Each experiment was independently conducted at least three times. **J** Effects of circEPB41L2 on the liver metastasis of Nude mice. The tumours of liver metastasis were shown using red arrows. Data were indicated as mean ± S.D. *p < 0.05, **p < 0.01, ***p < 0.001, ****p < 0.0001 versus vector controls.

circEPB41L2 was downregulated in NSCLC cell lines including the A549 and H23 cell lines (Fig. 2J), both of which were selected to explore the impact of circEPB41L2 on the progression of NSCLC. The siRNA targeting the circEPB41L2 backsplicing junction was constructed; its strong knockdown efficiency in A549 and H23 cells can be observed in Fig. S1. We noticed that circEPB41L2 overexpression severely suppressed the proliferation of A549 and H23 cells (Fig. 3A, B); while its knockdown dramatically enhanced cell viability of A549 and H23 cells (Fig. S1C, D). Moreover, circEPB41L2 overexpression obviously inhibited cell migration and invasion of A549 and H23 cells (Fig. 3C, D); while circEPB41L2 depletion markedly promoted these malignant phenotypes in A549 and H23 cells (Fig. S1E, F). Wound healing assay also demonstrated that circEPB41L2 overexpression inhibited the migration of A549 and H23 cells (Fig. 3E, F), while circEPB41L2 silencing facilitated the migration of A549 and H23 cells (Fig. S1G, H), suggesting that circEPB41L2 blocked NSCLC cell growth.

In vivo, circEPB41L2 overexpression inhibited tumour growth and decreased Ki67 staining of tumour tissues compared to that of controls (Fig. 3G, H). As shown in Fig. 3I, the firefly luciferase activity of metastatic lesions in the circEPB41L2 overexpression group was markedly reduced at day 28 (Fig. 3I). Similarly, the fewer and smaller foci of liver metastasis were observed in circEPB41L2 overexpression group (Fig. 3J). These data strongly suggest that circEPB41L2 supresses the tumour growth and metastasis of NSCLC in vitro and in vivo.

### circEPB41L2 binds to PTBP1 to enhance its ubiquitination and degradation

**Fig. 4:**
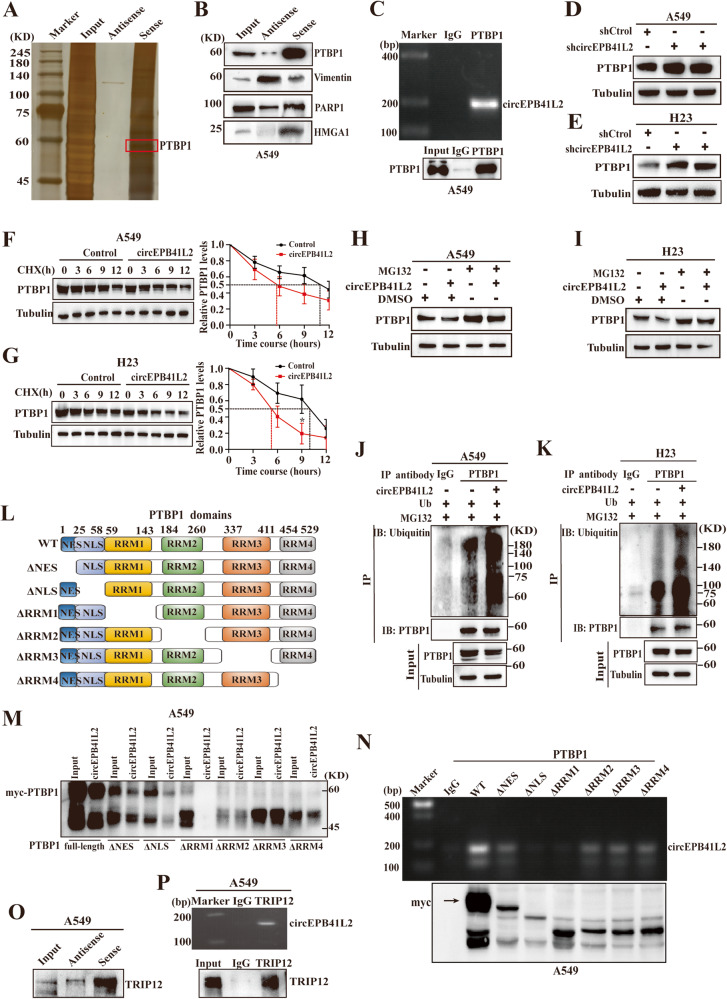
circEPB41L2 binds to PTBP1 to enhance its ubiquitination and degradation. **A** Biotinylated sense probe and antisense probe were incubated with A549 cell lysates for RNA pull-down assay. RNA-captured proteins were detected by silver staining and mass spectrometry. **B** circEPB41L2-captured proteins (PTBP1, Vimentin, PARP1 and HGMA1) with higher scores were identified by RNA pull-down and Western blotting assays using indicated antibodies in A549 cells. **C** RIP assay was performed utilising anti-PTBP1 antibody to determine the interaction between PTBP1 and circEPB41L2 in A549 cells. **D**, **E** The effect of circEPB41L2 knockdown on PTBP1 protein expression. A549 and H23 cells were transfected with shcircEPB41L2 or paired control. **F**, **G** The effect of circEPB41L2 knockdown on half-life of PTBP1 protein depicted by *t*_1/2_. A549 and H23 cells were transfected with circEPB41L2 or paired control, then treated with cycloheximide in indicated time. Data were indicated as mean ± S.D. *p < 0.05, versus controls. **H**, **I** Effects of circEPB41L2 on the protein levels of PTBP1. circEPB41L2-overexpressing A549 and H23 cells were treated with MG132 for 6 h. Protein expression were measured by Western blotting. **J**, **K** Effects of circEPB41L2 on PTBP1 ubiquitination. A549 and H23 cells were transfected with plasmids HA-ubiquitin and circEPB41L2 or vector after treatment with MG132 for 6 h to block protein degradation, then measured by immunoprecipitation and Western blotting. **L** A schematic diagram showing the different domains of PTBP1 and six mutants, including NES, NLS and RRM1-4. **M**, **N** The interactions of circEPB41L2 with PTBP1 motifs in A549 cells detected by RNA pull-down and RIP assay. Anti-myc antibody was used for immunoprecipitation. **O** TRIP12 was dropped by a circEPB41L2 sense RNA probe rather than an antisense RNA probe in A549 cells. Western blotting assay with anti-TRIP12 antibody was employed to detect the interaction of circEPB41L2 with TRIP12. **P** The interaction of circEPB41L2 with TRIP12 in A549 cells. Each experiment was independently conducted at least three times.

Gene ontology analysis indicated dominant functions of downregulated circRNAs as protein binding partners (Fig. S2A). circEPB41L2 RNA pull-down assay indicated that in the circEPB41L2 probe lane, multiple protein bands were enriched at ~40–100 kDa, demonstrating the interactions of multiple proteins with circEPB41L2 (Fig. 4A and Table S1); therefore, four oncogenic proteins (PTBP1, Vimentin, PARP1 and HMGA1) were selected to confirm their interactions with circEPB41L2. Indeed, PTBP1, Vimentin, PARP1 and HMGA1 were pulled down by the circEPB41L2 sense RNA probe in A549 cells, and PTBP1 exhibited stronger binding to circEPB41L2 (Fig. 4B). A subsequent RIP assay with the PTBP1 antibody showed increased enrichment of circEPB41L2 in PTBP1 sediments in A549 cells (Fig. 4C), validating the interaction between circEPB41L2 and PTBP1.

Published work has shown that the PTBP1 can regulate the alternative splicing and biogenesis of circRNAs by functioning as a trans-regulation factor [19, 30, 31]. Unexpectedly, our data indicated that neither PTBP1 depletion nor PTBP1 overexpression significantly altered circEPB41L2 expression in A549 and H23 cells (Fig. S2B–E). Similarly, neither circEPB41L2 depletion nor circEPB41L2 overexpression significantly affected PTBP1 mRNA expression (Fig. S2F–I). These overexpression and knockdown efficiencies were in Fig. S2J–M.

Intriguingly, circEPB41L2 knockdown enhanced PTBP1 protein expression in A549 and H23 cells (Fig. 4D, E). Subsequently, the effects of circEPB41L2 on PTBP1 protein stability were verified by chase experiments using cycloheximide (CHX) to block protein synthesis and MG132 to inhibit the proteasome pathway. As expected, circEPB41L2 overexpression rapidly shortened the half-life (*t*_1/2_) of PTBP1 in A549 and H23 cells treated with CHX (Fig. 4F, G). Moreover, circEPB41L2 accelerated the protein degradation of PTBP1 in A549 and H23 cells after exposure to MG132 (Fig. 4H, I), indicating that circEPB41L2 was involved in regulating the proteasome degradation of PTBP1. Given that our gene ontology analysis predicted that ubiquitin ligase activity was linked to the functions of downregulated circRNAs (Fig. S2N), we sought to explore the impact of circEPB41L2 on PTBP1 ubiquitination. Indeed, circEPB41L2 overexpression facilitated PTBP1 ubiquitination in A549 and H23 cells (Fig. 4J, K). These findings demonstrate that circEPB41L2 could mediate PTBP1 degradation.

Next, we performed RNA pull-down and RIP assays with deletion mutants of PTBP1 to elucidate the region required for PTBP1 interaction with circEPB41L2 (Fig. 4L). The results indicated that RRM1 and NLS motif mutations abolished the association of circEPB41L2 with PTBP1 in A549 cells (Fig. 4M, N), confirming the direct interaction of circEPB41L2 with PTBP1 via the RRM1 and NLS motifs. Furthermore, we predicted the secondary structures of different circEPB41L2 variants by RNAfold [32], and two variants were identified (Fig. S3A, B). Certain PTBP1-targeting pyrimidine-rich motifs (e.g., UCUU, UCUUC, UCUCU) were enriched in the 3–4 hairpin regions of the stem-loops structures of the second variant (Fig. S3B, C), further suggesting the interaction between circEPB41L2 and PTBP1. The NLS domain of PTBP1 is reportedly initiated from amino acids 45 of PTBP1, and RRM1 covers a segment of PTBP1 from amino acids 59 to 143 of PTBP1 [33]. The N‐terminal part and RRM1 motif of PTB are responsible for interaction and posttranslational modifications, including distinct phosphorylation and acetylation by the RRM1-RNA complex [33–35]. Based on our data and previous studies, we speculate that the binding of circEPB41L2-RRM1 may contribute to PTBP1 ubiquitination.

Subsequently, we searched for an E3 ubiquitin protein in response to PTBP1 ubiquitination among circEPB41L2-dropped proteins, and only the E3 ubiquitin-protein ligase thyroid hormone receptor interacting protein 12 (TRIP12) was identified. The results of RNA pull-down and RIP assays indicated that TRIP12 was recognised by the circEPB41L2 sense RNA probe in A549 cells (Fig. 4O), and circEPB41L2 was identified in the TRIP12-labelled lane by the TRIP12 antibody (Fig. 4P), approving the direct interaction of circEPB41L2 with TRIP12. Several studies have indicated that TRIP12 modulates the stability of its substrates by adding polyubiquitin chains to promote their degradation [36–39]. We speculated that the E3 ubiquitin ligase TRIP12 might control the ubiquitination and degradation of PTBP1.

### TRIP12 is responsible for the ubiquitination and degradation of PTBP1

**Fig. 5:**
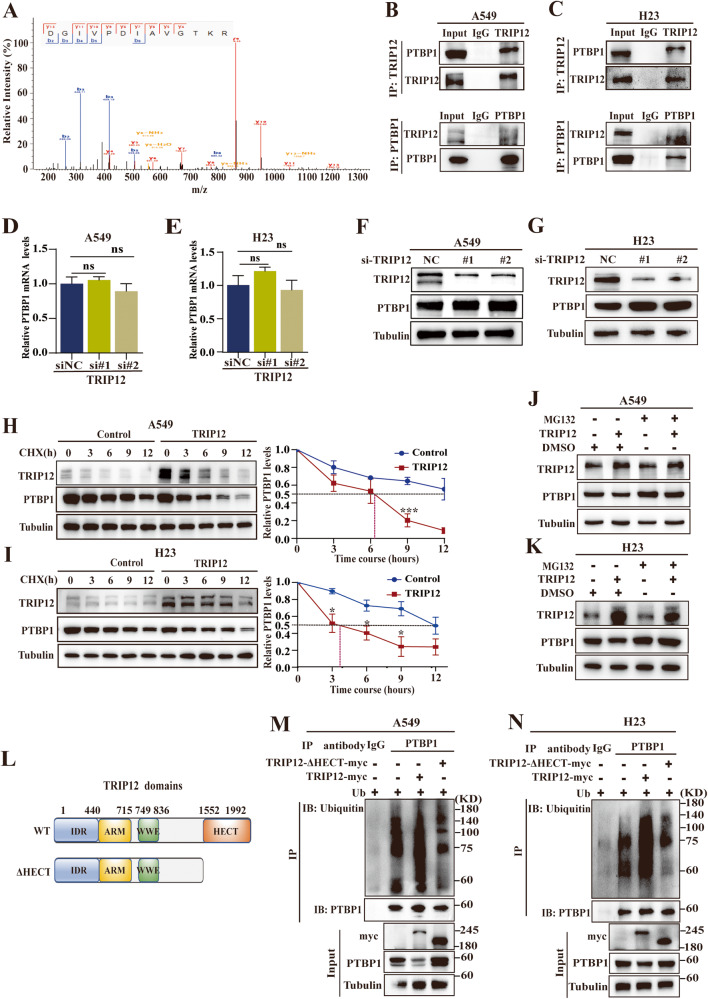
TRIP12 is responsible for the ubiquitination and degradation of PTBP1. **A** The peptide of PTBP1 was detected by MS analysis in TRIP12-overexpressing A549 cells. **B**, **C** TRIP12-PTBP1 interactions at endogenous levels in A549 and H23 cells determined by reciprocal coimmunoprecipitation. Up: PTBP1 was enriched in lysates from TRIP12-immunoprecipitated A549 and H23 cells. Down: TRIP12 was obtained in PTBP1-immunoprecipitated lysates from A549 and H23 cells. **D**, **E** The effects of TRIP12 depletion on PTBP1 mRNA expression in A549 and H23 cells. **F**, **G** The effects of TRIP12 knockdown on PTBP1 protein expression in A549 and H23 cells. **H**, **I** The effects of TRIP12 overexpression on the *t*_1/2_ of PTBP1 in A549 and H23 cells treated with cycloheximide in different time points. **J**, **K** The effects of TRIP12 on the protein levels of PTBP1 in a manner that depends on proteasomal activity. TRIP12 was transfected in A549 and H23 cells with or without MG132 for 6 h, then measured by Western blotting. **L** A schematic diagram showing the different domains of TRIP12-myc and its HECT deletion form (ΔHECT). **M**, **N** TRIP12 was responsible for PTBP1 ubiquitination. A549 and H23 cells were transfected with HA-ubiquitin plasmid and TRIP12-myc plasmid or TRIP12-ΔHECT-myc mutant for 48 h, then cells were treated with MG132 for 6 h to restrict protein degradation. The protein expression was measured by Western blotting. Each experiment was independently conducted at least three times.

PTBP1 was the candidate that interacted with TRIP12 in MS analysis (Fig. 5A). Subsequent results indicated that PTBP1 was enriched in lysates from TRIP12-immunoprecipitated A549 and H23 cells; similarly, TRIP12 was detected in lysates from PTBP1-immunoprecipitated A549 and H23 cells (Fig. 5B, C), suggesting the interaction between TRIP12 and PTBP1.

Additionally, TRIP12 silencing in A549 and H23 cells slightly altered the PTBP1 mRNA levels (Fig. 5D, E) but enhanced its protein levels (Fig. 5F, G). TRIP12 overexpression dramatically shortened the *t*_1/2_ of the PTBP1 protein in A549 and H23 cells treated with CHX (Fig. 5H, I) and accelerated PTBP1 protein degradation, which was reversed by MG132 treatment (Fig. 5J, K). These results suggest that TRIP12-triggered downregulation of PTBP1 requires the activity of the 26S proteasome.

Next, TRIP12-induced PTBP1 ubiquitination was examined using TRIP12-WT-myc or TRIP12-ΔHECT-myc, the TRIP12 HECT domain-deleted mutant (Fig. 5L). The results indicated that TRIP12 overexpression significantly stimulated PTBP1 ubiquitination (Fig. 5M). A similar result was observed in H23 cells (Fig. 5N). The domain homologous to the E6-associated protein carboxyl terminus (HECT domain) of TRIP12 has been reported to be required for its ubiquitin activity [36]. The HECT domain-deletion mutant of TRIP12-myc was used to determine the precise regulatory effect of TRIP12 on PTBP1 ubiquitination (Fig. 5L). As expected, overexpressed TRIP12 rather than catalytically dead TRIP12 with its HECT domain deleted enhanced PTBP1 ubiquitination levels (Fig. 5M, N). Collectively, these findings provide convincing evidence that TRIP12 promotes PTBP1 ubiquitination and degradation via HECT domain.

### circEPB41L2 is critical for the PTBP1 ubiquitination and degradation mediated by TRIP12

**Fig. 6:**
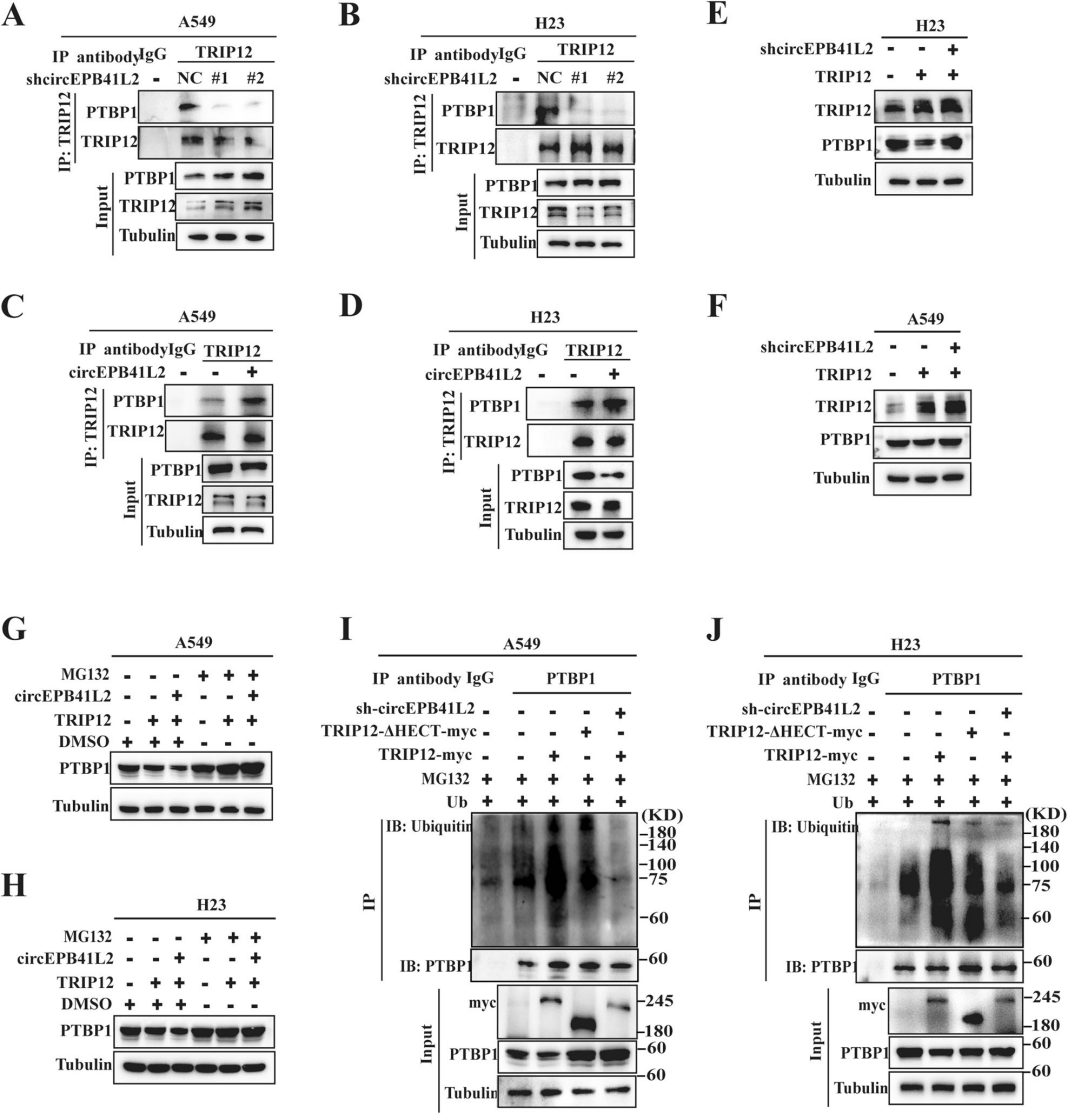
circEPB41L2 is critical for the PTBP1 ubiquitination and degradation mediated by TRIP12. **A**, **B** The effects of circEPB41L2 knockdown on the interaction between TRIP12 and PTBP1 in A549 and H23 cells. **C**, **D** The effects of circEPB41L2 overexpression on endogenous interaction between TRIP12 and PTBP1 in A549 cells and H23 cells. Anti-TRIP12 was employed for immunoprecipitation. Antibodies against TRIP12, PTBP1 and Tubulin were used for Western blotting. **E**, **F** The effects of circEPB41L2 depletion on TRIP12-induced PTBP1 expression in H23 and A549 cells. Cells were transfected with TRIP12 or vector in the presence or absence depleted circEPB41L2. **G**, **H** The effects of circEPB41L2 on TRIP12-induced protein degradation in a manner that depends on proteasomal activity. TRIP12-expressed A549 and H23 cells were transfected with control or circEPB41L2 with or without MG132, then measured by Western blotting. **I**, **J** The effects of circEPB41L2 on TRIP12-induced PTBP1 ubiquitination. A549 and H23 cells were transfected with plasmids HA-ubiquitin and TRIP12-WT-myc or TRIP12-ΔHECT-myc, or TRIP12-WT-myc and sh-circEPB41L2, which was treated with MG132 for 6 h to block protein degradation. Each experiment was conducted independent three times.

Our results confirmed the binding of circEPB41L2 with TRIP12 and PTBP1 (Fig. 4). We then explored the roles of circEPB41L2 in TRIP12-PTBP1 interactions. The results demonstrated that circEPB41L2 knockdown obviously reduced the interaction of TRIP12 with PTBP1 compared to the controls in A549 and H23 cells (Fig. 6A, B). circEPB41L2 overexpression yielded the opposite result (Fig. 6C, D). Markedly, circEPB41L2 depletion in TRIP12-overexpressing H23 cells contributed to an enhanced recovery of PTBP1 protein expression compare to the decreased PTBP1 levels (Fig. 6E). These results were also observed in A549 cells (Fig. 6F). Correspondingly, circEPB41L2 cotransfection enhanced TRIP12-induced PTBP1 protein degradation in MG132-treated A549 and H23 cells (Fig. 6G, H), suggesting that circEPB41L2 is required for the interaction of PTBP1 with TRIP12.

We observed that TRIP12 overexpression dramatically increased the PTBP1 ubiquitination levels, which were reduced by TRIP12 HECT domain depletion, and sharply attenuated by circEPB41L2 depletion (Fig. 6I). Similar results were obtained in H23 cells (Fig. 6J), demonstrating the capability of circEPB41L2 to enhance the TRIP12-stimulated PTBP1 ubiquitination and degradation.

### circEPB41L2 blocks PTBP1-induced progression and metastasis of NSCLC

**Fig. 7:**
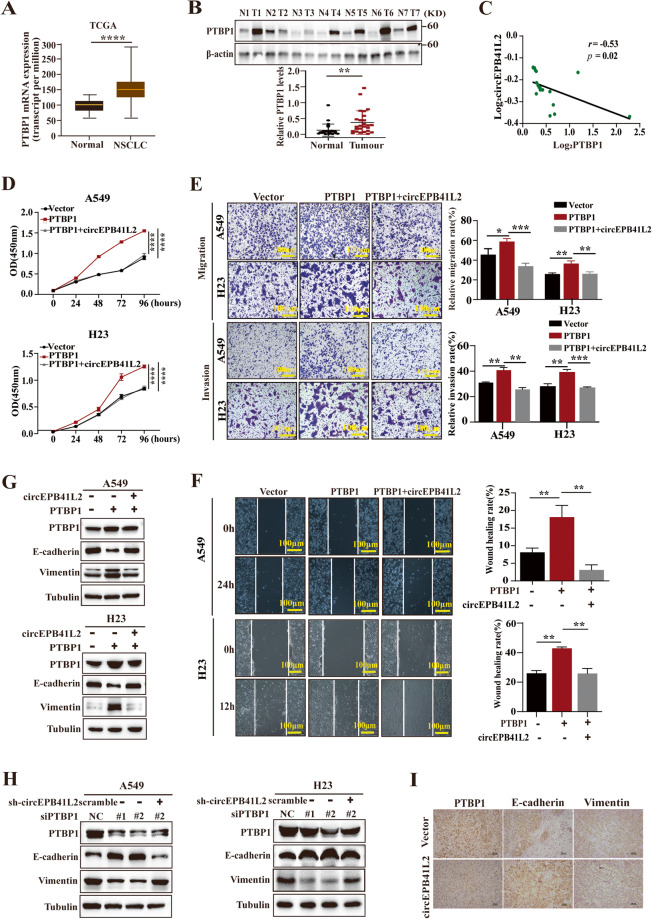
circEPB41L2 blocks PTBP1-induced progression and metastasis of NSCLC. **A** PTBP1 mRNA expression in tumour tissues and adjacent control tissues of NSCLC from TCGA datasets. **B** PTBP1 protein expression in tumour and adjacent control tissues of NSCLC. **C** The association of PTBP1 protein expression with circEPB41L2 levels in NSCLC tissues. **D** Effects of circEPB41L2 on PTBP1-induced cell proliferation of A549 and H23 cells, which was measured by CCK8 assays. **E** Effects of circEPB41L2 on PTBP1-induced cell migration and invasiveness of A549 and H23 cells, which was detected by transwell assays. **F** Effects of circEPB41L2 on PTBP1-induced cell migration of A549 and H23 cells, which was tested by wound-healing assays. **G** Effects of circEPB41L2 overexpression on the protein expression of PTBP1, E-cadherin and Vimentin in PTBP1 overexpressed A549 and H23 cells with or without circEPB41L2. **H** Effects of circEPB41L2 knockdown on the protein expression of PTBP1, E-cadherin and Vimentin in PTBP1 depleted A549 and H23 cells with or without depleted circEPB41L2. Protein expression was measured by Western blotting. **I** Protein expression of PTBP1, E-cadherin and Vimentin on circEPB41L2-overexpressed tumour tissues of nude mice xenografts, which was detected by IHC assay. Each experiment was independently conducted at least three times. Data were indicated as mean ± S.D. *p* value was determined by a two-tailed paired Student’s *t* test. *p < 0.05, **p < 0.01, ***p < 0.001, ****p < 0.0001 versus controls.

Our previous work exhibited that PTBP1 knockdown can suppress the development and metastasis of renal cell carcinoma [28]. Currently, employing the datasets from TCGA and Clinical Proteomic Tumor Analysis Consortium (CPTAC, http://ualcan.path.uab.edu/analysis.html), we demonstrated that PTBP1 mRNA and protein levels were markedly elevated in NSCLC tissues compared to adjacent controls (Fig. 7A, B), which was negatively associated with circEPB41L2 levels (Fig. 7C). Furthermore, PTBP1 overexpression significantly promoted the proliferation and the migration of A549 and H23 cells, cotransfection clearly blocked these phenomena (Fig. 7D, E). Furthermore, circEPB41L2 cotransfection restricted PTBP1-induced the migration of A549 and H23 cells conducted by wound healing assays (Fig. 7F).

Moreover, PTBP1 overexpression facilitated epithelial-mesenchymal transition (EMT) by reducing E-cadherin expression but enhancing Vimentin expression in A549 and H23 cells, and these effects were abolished by circEPB41L2 cotransfection (Fig. 7G). Instead, PTBP1 knockdown elevated E-cadherin expression but decreased Vimentin expression. This was clearly reversed by cotransfection with depleted circEPB41L2 (Fig. 7H). Furthermore, IHC assay results exhibited that E-cadherin expression was dramatically elevated but expression of PTBP1 and Vimentin was obviously reduced in circEPB41L2-overexpressing tumour tissues compared to controls (Fig. 7I), suggesting that circEPB41L2 inhibits NSCLC growth and metastasis by blocking PTBP1-mediated Vimentin activation and E-cadherin inactivation in vitro and in vivo.

### circEPB41L2 restricts PTBP1-induced aerobic glycolysis in NSCLC via PTBP1/PKM2 axis

**Fig. 8:**
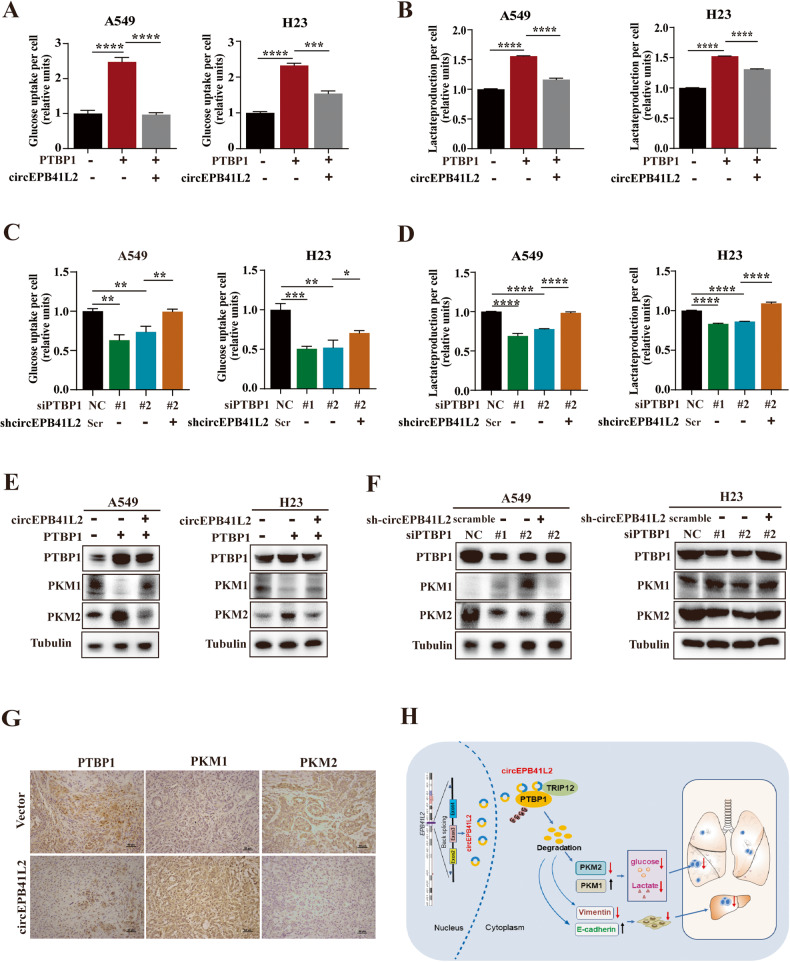
circEPB41L2 restricts PTBP1-induced aerobic glycolysis in NSCLC via PTBP1/PKM2 axis. **A** Effects of circEPB41L2 overexpression on PTBP1-induced glucose uptake in A549 and H23 cells. **B** Effects of circEPB41L2 overexpression on PTBP1-induced lactate production in A549 and H23 cells. **C** Effects of circEPB41L2 knockdown on the glucose uptake in A549 and H23 cells with PTBP1 depletion. **D** Effects of circEPB41L2 knockdown on the lactate production in A549 and H23 cells with PTBP1 depletion. A549 and H23 cells were cotransfected depleted PTBP1 with or without depleted circEPB41L2 for 48 h, then the levels of glucose uptake or lactate production were measured using the corresponding kits. Scr: scramble. **E** Effects of circEPB41L2 overexpression on protein expression of PTBP1, PKM1 and PKM2 in PTBP1 overexpressed A549 and H23 cells with or without circEPB41L2. **F** Effects of circEPB41L2 knockdown on protein expression of PTBP1, PKM1 and PKM2 in PTBP1 depleted A549 and H23 cells with or without depleted circEPB41L2. Protein expression was measured by Western blotting. **G** The protein expression of PTBP1, PKM1 and PKM2 in circEPB41L2-overexpressed tumour tissues of nude mice xenografts, which was detected by IHC assay. Each experiment was independently performed at least three times. Data were indicated as mean ± S.D. *p < 0.05, **p < 0.01, ***p < 0.001, ****p < 0.0001 versus controls. **H** Schematic of circEPB41L2 blocks aerobic glycolysis, progression and metastasis of NSCLC through TRIP12-mediated PTBP1 ubiquitylation.

PTBP1 is reportedly to be participated in metabolic reprogramming in cancer progression due to controlling biogenesis of Pyruvate kinase M2 subtype (PKM2), a crucial member of pyruvate kinase which is required for the lactate production during metabolic reprogramming in cancer [40, 41]. The function of circRNA in PTBP1-triggered metabolic reprogramming in NSCLC has not been full investigated, however. Our results indicated that PTBP1 overexpression dramatically enhanced glucose uptake (Fig. 8A) and lactate production in A549 and H23 cells (Fig. 8B), and this could be clearly inhibited by circEPB41L2 cotransfection (Fig. 8A, B). Moreover, PTBP1 knockdown reduced glucose uptake (Fig. 8C) and lactate production (Fig. 8D) in A549 and H23 cells, which could be reversed by circEPB41L2 depletion (Fig. 8C, D). Importantly, PTBP1 overexpression reduced the PKM1 levels but increased the PKM2 levels, and these results were obviously reversed by circEPB41L2 cotransfection in A549 and H23 cells (Fig. 8E). Instead, PTBP1 silencing increased PKM1 levels but decreased PKM2 levels in A549 and H23 cells, and cotransfection with depleted circEPB41L2 led to the opposite phenomenon (Fig. 8F). IHC assay results demonstrated that PKM1 expression was dramatically elevated but PKM2 expression was obviously reduced in circEPB41L2-overexpressing tumour tissues compared to paired controls (Fig. 8G). These findings verify that circEPB41L2 blocks aerobic glycolysis in NSCLC via PTBP1/PKM2 axis.

## Discussion

Numerous studies have demonstrated the sponge-like function of circRNAs in absorbing microRNAs and regulating RNA expression [12–14]. Here, using RNA-seq of LUAD and adjacent normal tissues, we identified a downregulated circRNA named circEPB41L2 that was associated with unfavourable prognosis in patients with NSCLC. Mechanistically, circEPB41L2 blocked PTBP1 expression by binding to the RRM1 domain of PTBP1 and interacting with the E3 ubiquitin ligase TRIP12 to promote PTBP1 ubiquitination and degradation, blocking the PTBP1-induced PKM2 activation and PKM1 inactivation as well as Vimentin activation and E-cadherin inactivation, thus inhibiting aerobic glycolysis, development and metastasis of NSCLC (Fig. 8H). Our findings highlight the circEPB41L2-dependent mechanism that modulates the “Warburg Effect” to inhibit the progression and metastasis of NSCLC by binding to the crucial motif of an RNA-binding protein for ubiquitination via a novel ubiquitin ligase, thus expanding the understanding of the multifaceted functions of circular RNAs in NSCLC progression.

Recently, the discovery of circRNA-relevant protein interactions such as RBP-mediated circRNA biogenesis has attracted increasing attention [22–24]. RBPs function as either activators or repressors that govern circRNA circularisation due to interplay with specific motifs in flanking introns (“GGT-rich” motifs) [42]. Unexpectedly, our results suggest that overexpression and depletion of PTBP1, an RNA binding protein, failed to significantly alter circEPB41L2 expression in NSCLC cells. However, circEPB41L2 overexpression dramatically reduced PTBP1 protein expression instead of mRNA expression, which could be reversed after treatment with MG132, a specific proteasome inhibitor. These findings suggest a novel function of circEPB41L2 that involves the regulation of PTBP1 stability via the proteasome degradation pathway. Importantly, we discovered that the interaction between circEPB41L2 and PTBP1 depends on the RRM1 and NLS motifs of PTBP1. The N‐terminal part and RRM1 motif of PTBP1 are reportedly responsible for its interaction and posttranslational modifications, including distinct phosphorylation and acetylation by formulation of the RRM1-RNA complex [33–35]. We reveal that the RRM1 and NLS motifs of PTBP1 are indispensable for the interaction of circEPB41L2 with PTBP1, and a number of UCUUU loop sequences are observed in the secondary structure of circEPB41L2, demonstrating that the interaction of circEPB41L2 with PTBP1 through its N-terminal RRM1 may contribute to the exposure of the ubiquitination region for subsequent contact and ubiquitination of TRIP12. A detailed mechanism is required for further elucidation.

TRIP12, a novel E3 ubiquitin ligase, has been documented to be responsible for the ubiquitin-mediated degradation of several proteins including FBW7 and nuclear factor of activated T cell cytoplasmic 1 (NFATc1) [36–39], and its HECT domain is crucial for ubiquitin-mediated degradation of NFATc1, a PD-1 transcription activator [36]. Khan et al. revealed that the HECT-domain in the E3 ubiquitin ligase TRIP12 is indispensable for proteasomal degradation of the tumour suppressor FBW7 to sensitise cells to Taxol chemotherapy [37]. Our findings demonstrate that TRIP12-initiated PTBP1 ubiquitination and degradation depend on its HECT domain-catalysed activity. Importantly, this regulation requires the involvement of circEPB41L2 as a scaffold, suggesting that circEPB41L2 is important for governing the protein stability of PTBP1 by integrating with TRIP12, an E3 ubiquitin ligase. Recent evidence indicated that circNDUFB2 was capable of suppressing tumorigenicity and metastasis of NSCLC by functioning as a protein scaffold to bind to KH domains of the IGF2BP protein and recruit of TRIM25, an E3 ubiquitin ligase, thus accelerating IGF2BP degradation [16]. Intriguingly, we discovered that the PTBP1 RRM1-circRNA complex may favour the ubiquitination of the E3 ubiquitin ligase TRIP12, which may expand the understanding of circRNA function linking RNA secondary structure.

Tumour cells prefer glycolytic pathways to oxidative phosphorylation, which converts glucose mainly to lactate. PKM2 is a pivotal glycolytic enzyme that facilitates lactate production and metabolic reprogramming to promote cancer growth, invasion and migration [40, 43]. PKM2 biogenesis tightly depends on splicing switch induced by PTBP1 expression [40, 41]. By recognition of the poly(U) tracts in the 3’ untranslated regions (UTRs) of mRNA, PTBP1 enhances the alternative mRNA splicing of MEIS2-L and PKM2 variants to facilitate the lymphatic metastasis and proliferation of bladder cancer [43]. Aberrant PTBP1 expression also accelerates the oncogenic splicing switch in pyruvate kinase from splice variant PKM1 towards PKM2, triggering energy metabolism remodelling from oxidative phosphorylation to aerobic glycolysis [40, 43]. Our findings indicate that PTBP1 promotes glucose uptake capacity and lactate levels in NSCLC cells via elevating PKM2 expression but attenuating PKM1 expression. Importantly, this can be impaired by circEPB41L2, presenting novel evidence that circEPB41L2 is linked to aerobic glycolysis, a hallmark of metabolic reprogramming in human cancers. In addition to acting as a blocker of the PTBP1-mediated switch of PKM1 to PKM2, circEPB41L2 also acts as an inhibitor of PTBP1-stimulated Vimentin activation and E-cadherin inactivation to limit NSCLC development and metastasis. Our findings provide novel insights into the ability of circular RNA (circEPB41L2) that participates in glycolytic metabolism and epithelial-mesenchymal transition to inhibit NSCLC development and metastasis.

Collectively, our data reveal a novel mechanism by which circEPB41L2 regulates glycolytic metabolism and EMT to inhibit NSCLC development and metastasis via binding to the RRM1 domain, the crucial motif of an RNA-binding protein for the ubiquitination by TRIP12, which reduces PKM2 and Vimentin expression but enhances PKM1 and E-cadherin expression. Moreover, the analysis of the secondary structure of circRNAs linked to proteins may be a new strategy to identify novel biomarkers of NSCLC.

## Material and methods

### Tissue specimens

Fifty pairs of primary NSCLC tissues and adjacent tissues were collected in the Affiliated Hospital of Xuzhou Medical University (Xuzhou, China) from January 2018 to August 2021(XYFY2018-KL023-01). Of which, six paired cancer and adjacent cancer tissues were employed for ribosomal RNA-depleted RNA seq. The details of clinical characteristics of all subjects were summarised in Table 1.

The subjects were obtained from the same ethnic group and the same criteria: (1) The NSCLC diagnoses were certified by two pathologists. (2) All subjects of NSCLC did not receive any treatment before surgery. (3) None of the patients was died during this study. (4) No history of other synchronous malignancies.

### RNA sequencing

Total RNA was extracted from six pairs of freshly frozen LUAD tissues and matched controls, following treatment with NEB Next rRNA Depletion Kit (New England Biolabs, Inc., Massachusetts, USA) to remove ribosomal RNA (rRNA) for the degradation of linear RNAs and enrichment of circRNAs. RNA libraries were constructed using NEBNext® Ultra™ II Directional RNA Library Prep Kit (New England Biolabs, Inc., Massachusetts, USA) according to the manufacturer’s instructions. Subsequent transcriptome on an Illumina HiSeq 4000 was conducted by the Cloud-Seq Biotech Ltd. Co. (Shanghai, China). Significant differential expressed circRNAs were screened by fold change >2 or <−2 and *p* value < 0.05.

### Cell lines and cultivation

Human NSCLC cell lines (A549, H23, H1299 and H292 cells) and human bronchial epithelial cell line (BEAS-2B) and HEK-293T cells were purchased from ATCC and authenticated by short tandem repeat profiling by Genewiz, CN or ATCC. HEK293T cells were grown in Dulbecco’s modified DMEM supplemented with 10% fetal bovine serum. The other cell lines were cultured in RPMI 1640 (Life Technologies, Grand Island, NY, USA) containing 10% fetal bovine serum (Gibco, Grand Island, NY, USA). All cells were all cultured at 37 °C with 5% CO_2_ and free from mycoplasma contamination. The details of cell culture mediums were listed in Table S2.

### Plasmids, reagents and small interfering RNAs

The sequences of circEPB41L*2* were inserted into the pLC5-ciR (GFP) circRNA vector (Geneseed Biotech Co, Guangzhou, China) to construct an overexpressed plasmid. The details of plasmids containing myc-PTBP1, TRIP12-WT-myc and TRIP12-ΔHECT-myc, pCDNA-HA-Ub-WT for ubiquitination assay and reagents including MG132 and Cycloheximide (CHX) for exploring of protein stability and siRNAs or shRNAs particularly targeted circEPB41L2, or PTBP1 or TRIP12 were presented in Table S2.

### qRT-PCR, Sanger sequencing, gel electrophoresis, immunohistochemistry (IHC) and Western blotting

qRT-PCR, RT-PCR, Sanger sequencing, gel electrophoresis, immunohistochemistry (IHC) and Western blotting were conducted as previously reported [18, 44, 45]. More details of antibodies and primers used in our study were listed in Tables S2 and S3.

### RNase R treatment

Two micrograms of total RNA were cultivated for 30 min at 37 °C with or without 3 U/μg RNase-R (Epicentre Technologies, Madison, WI, USA), and measured by qRT-PCR.

### Actinomycin D assays

In total, 5 × 10^5^ cells were seeded in six-well plates. After 24 h, cells were treated with 3 μg/ml Actinomycin D (Sigma, USA) and collected at different time points. The RNA levels were determined by qRT-PCR and normalised to the values measured in the mock treatment group (the 0 h group).

### RNA nucleus-cytoplasm separation

The nuclear and cytoplasmic fractions of cells were separated using the PARIS™ kit (Invitrogen™, USA) according to the manufacturer’s instructions. In total, 1 × 10^6^ cells were resuspended on ice utilising cold cell fractionation buffer for 5 min, and then centrifuged at 500 × *g* for 5 min. The supernatant was collected; nuclear fractionation was cultured in cold cell disruption buffer, followed by RNA determination with qRT-PCR.

### Cellular proliferation, cell migration and invasion assays

Cell Counting Kit-8 (CCK-8) assays were performed as previously reported [44]. Invasion assays were conducted utilising modified Transwell inserts (Corning, Inc., Corning, NY, USA) based on the manufacturer’s instruction.

### RNA pull-down assay

The 3’ biotin-labelled oligonucleotide probe targeting the junction site of circEPB41L2 was synthesised by the Ruibo Biotechnology Co., LTD (Guangzhou, China). circRNA pull-down assay was conducted as previous literature with slight modification [16]. In brief, 2 × 10^7^ cells were washed in ice-cold PBS, lysed in 1000 μl buffer mixed with cocktail of protease inhibitor (Roche, Swit), and RNase inhibitor (Beyotime, Shanghai, China) incubated for 60 min at room temperature, then cultured with 100 pmol biotinylated circEPB41L2 (sense) or corresponding complementary probes (antisense) at 4 °C overnight. Eighty microliters of streptavidin magnetic beads (HY-K0208, MCE, USA) were added to each binding reaction of cell lysates and incubated for 6 h at 4 °C; then beads were washed for three times and boiled in SDS buffer. The retrieved proteins were employed for Western blotting assay or MS.

### circRNA binding protein immunoprecipitation (circRIP) assay

circRIP assay was implemented following the manufacturer’s instructions. Three micrograms of varied antibodies were coated with protein A/G agarose beads and incubated with cell lysates at 4 °C overnight. Coprecipitated RNA was examined by qRT-PCR. Details of Reagents were shown in Table S2.

### Immunoprecipitation (IP) and ubiquitination assays

IP assay was conducted followed previous reports [36, 45]. More details of antibodies used in our study were listed in Table S2. For PTBP1 protein ubiquitination, the lysates were extracted from cells cotransfected circEPB41L2 or sh-circEPB41L2 with HA-ubiquitin, or cells cotransfected TRIP12-WT-myc or TRIP12-ΔHECT-myc with HA-ubiquitin and myc-PTBP1 plasmids. These cells were treated with 10 µM MG-132 for 6 h before harvest, cultured with anti-PTBP1, following protein A/G PLUS-Agarose beads, and then determined by Western blotting.

### Animal work

Four- to six-week-old male BALB/c nude mice were obtained from Beijing Vital River Laboratory Animal Technology Co., Ltd. (Beijing, China). Six mice were randomly assigned to each group. A549 cells transfected with circEPB41L2 or vector plasmid were employed for the tumour growth assay in vivo. A549 cells (1 × 10^7^) were subcutaneously injected into the right flank of mice (*n* = 5 per group). Tumour volumes were calculated using the formula *V* = *a* × (*b* × *b*)/2. A tail vein metastasis model was performed in BALB/c nude mice. Stably infected circEPB41L2 and vector A549 cells with luciferase lentivirus were administered to nude mice. Luciferase activity was measured by bioluminescence imaging. Mice were sacrificed after 4 weeks. The tumour tissues were excised for further analyses.

### Glucose uptake, lactate production measurement

Cells were cultured in six-well plates for 48 h after depletion or overexpression of PTBP1 with or without circEPB41L2. Glucose assay kit (Shanghai Rongsheng Biotech, Shanghai, China) and lactate assay kit (#A019-1-1, Nanjing Jiancheng Bioengineering Institute, Nanjing, China) were obtained to examine the glucose uptake, lactate production following manufacturer’s instructions.

### Statistical analysis

Data were conducted with GraphPad Prism 8.0 (GraphPad Software, La Jolla, CA). The Circos (http://circos.ca/) was employed for characterisation of circRNAs in chromosomes from our RNA-seq. Student’s *t* test and one way ANOVA test were applied according to actual conditions. ROC analysis was used for evaluation diagnose potentiality of circRNA. *p* < 0.05 was considered as statistically significant.

### Supplementary information

Supplementary Figures

Supplementary Table

Full and uncropped western blots

## Data Availability

The datasets used or analysed during the current study are available from the corresponding author on reasonable request.
