# Supplementary material for: circEPB41L2 blocks the progression and metastasis in non-small cell lung cancer by promoting TRIP12-triggered PTBP1 ubiquitylation
Source: Cell Death Discov. 2024 Feb 10;10:72. doi: 10.1038/s41420-024-01836-4 (PMC10858955; doi:10.1038/s41420-024-01836-4)
Supplement: Supplementary file 1 — Supplementary Figures [file 41420_2024_1836_MOESM1_ESM.docx]

**Supplementary Figures**


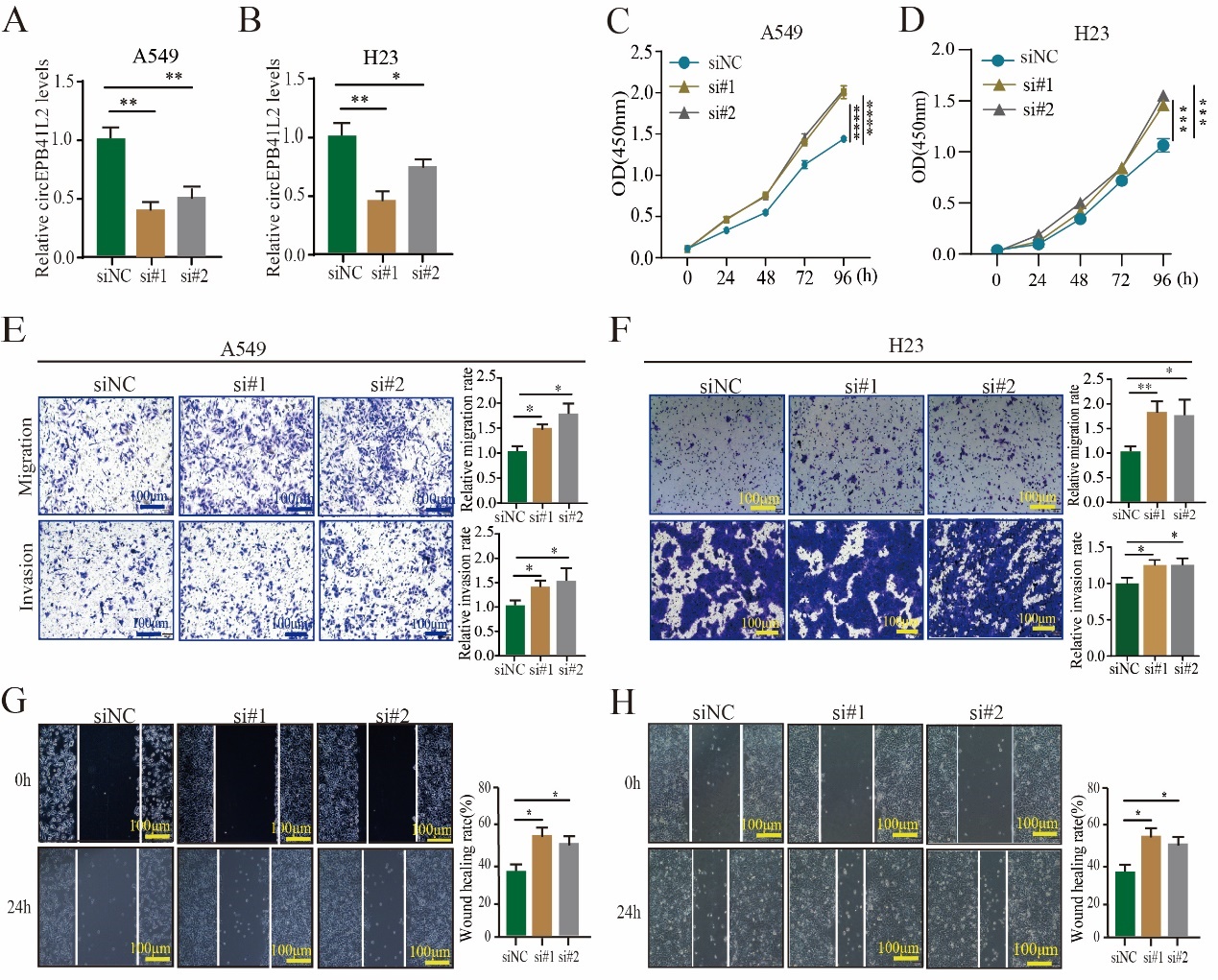


**Supplementary Figure 1.** **circEPB41L2 silencing promotes cell growth of NSCLC.** **A-B** The knockdown efficiencies of circEPB41L2 in A549 and H23 cells. **C-D** circEPB41L2 knockdown facilitated the proliferation inA549 and H23 cells measured by CCK8 assay. **E-F** circEPB41L2 knockdown accelerated migration and invasion in A549 and H23 cells determined by transwell assays. **G-H** The effects of circEPB41L2 knockdown on cell migration of A549 and H23 cells were further examined by wound-healing assays. Each experiment was independently conducted at least three times. Data was indicated as mean ± S.D. Statistical differences were analyzed using the unpaired *t* test. **p* < 0.05, ***p* < 0.01 versus paired controls.


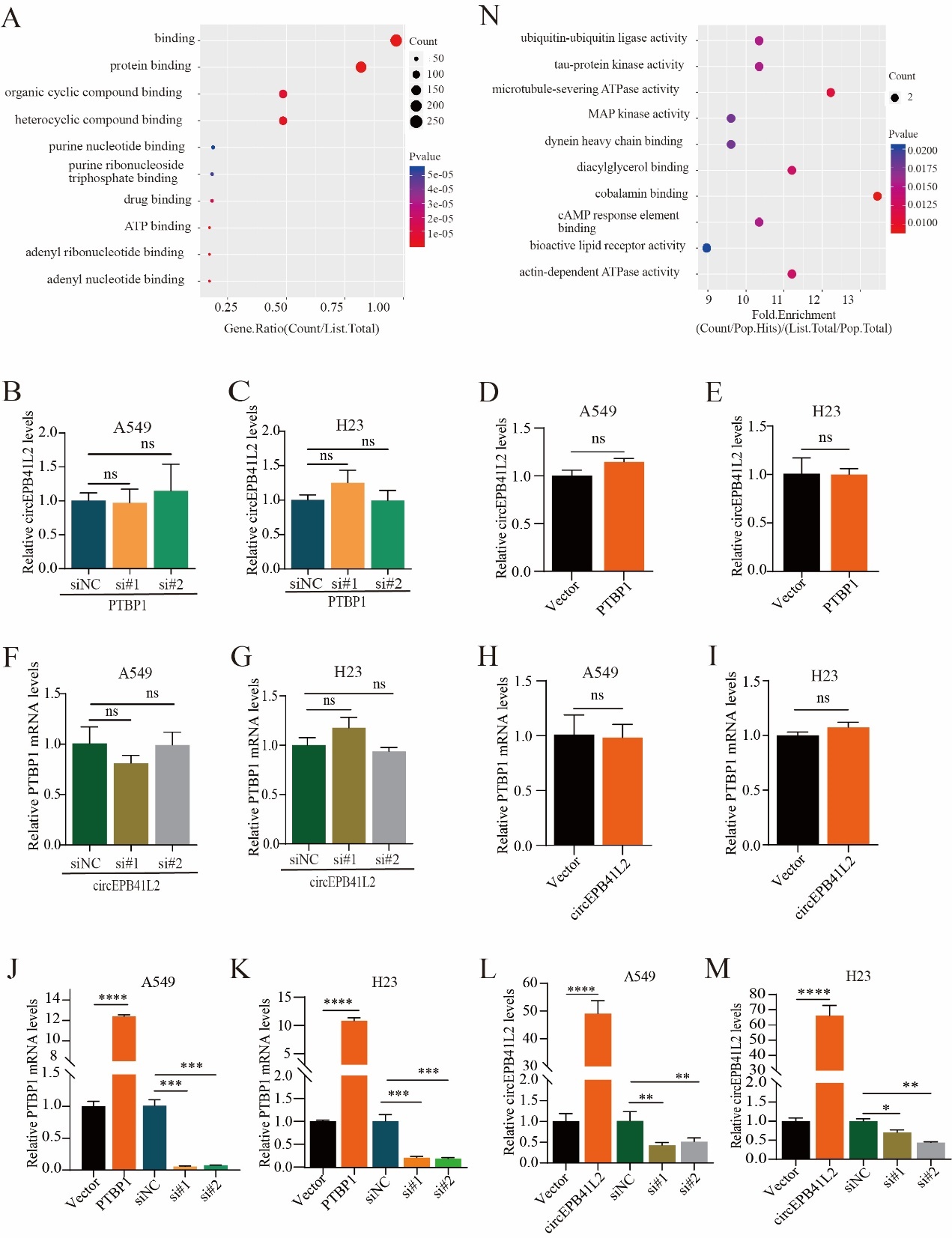


**Supplementary Figure 2. circEPB41L2 interacts with PTBP1.**

**A** Gene ontology analysis predicted dominant functions of downregulated circRNAs. **B-C** The effect of PTBP1 knockdown on circEPB41L2 levels. A549 and H23 cells were transfected with siPTBP1 or control. qRT-PCR assay was used to examine RNA levels. **D-E** The effect of PTBP1 overexpression on circEPB41L2 levels. A549 and H23 cells were transfected with PTBP1 or Vector. **F-G** The effect of circEPB41L2 silence on PTBP1 mRNA expression. A549 and H23 cells were transfected with sicircEPB41L2 or control. **H-I** The effect of circEPB41L2 overexpression on PTBP1 mRNA expression. A549 and H23 cells were transfected with circEPB41L2 or control. **J-K** The overexpression and knockdown efficiency of PTBP1 in A549 and H23 cells. **L-M** The overexpression and knockdown efficiencies of circEPB41L2 in A549 and H23 cells. **N** Gene ontology analysis predicted potential functions of downregulated circRNAs as protein partners. Data were indicated as mean ± S.D. Each experiment was independently conducted at least three times. **p* < 0.05, ***p* < 0.01, ****p* < 0.001, *****p* < 0.0001 versus matched controls.

**
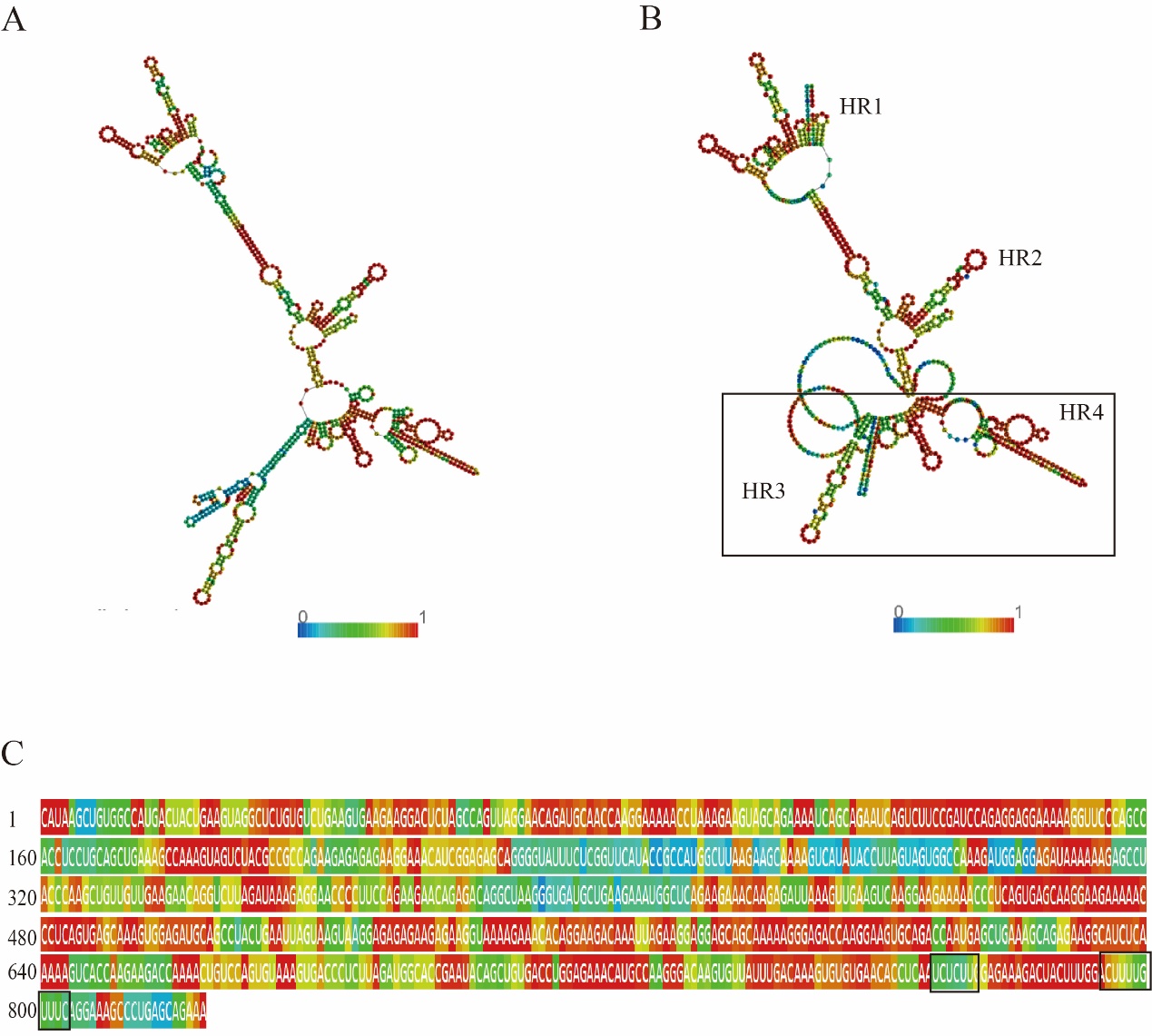
**

**Supplementary Figure 3. Prediction of the secondary structure of circEPB41L2 and the potentiality interacting with PTBP1.**

**A-B** Predicted secondary structures of two circEPB41L2 variants using RNAfold (http://rna.tbi.univie.ac.at/). Predictions were based on minimum free energy (MFE) and partition function. **C** The sequence of circEPB41L2. The block box denotes the PTBP1-targeting pyrimidine-rich motifs (e.g., UCUU, UCUUC, UCUCU) found in the sequence of circEPB41L2. Different colors indicated base-pairing probability. HR: hairpin region.
