# Supplementary material for: circEPB41L2 blocks the progression and metastasis in non-small cell lung cancer by promoting TRIP12-triggered PTBP1 ubiquitylation
Source: Cell Death Discov. 2024 Feb 10;10:72. doi: 10.1038/s41420-024-01836-4 (PMC10858955; doi:10.1038/s41420-024-01836-4)

Full and uncropped western blots for circEPB41L2

Figure4A

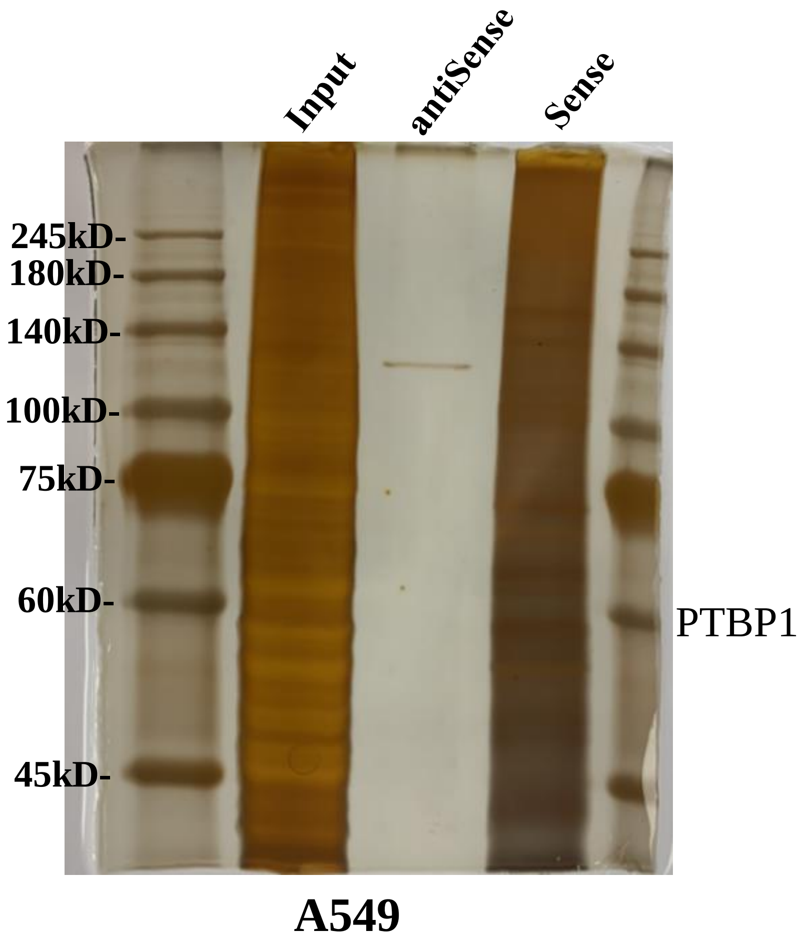

Figure4B

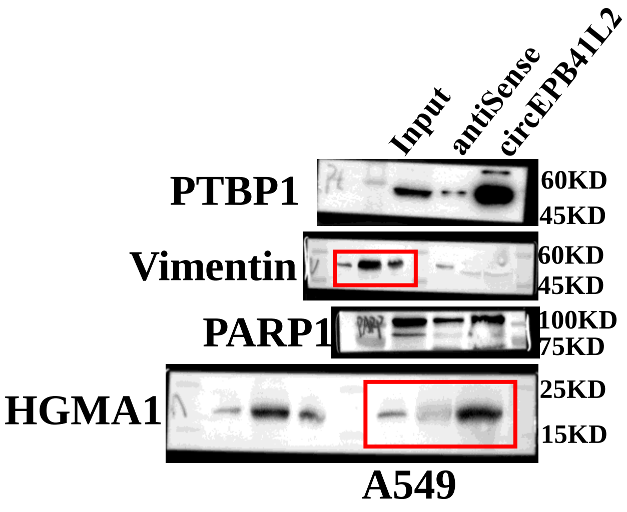

Figure4C

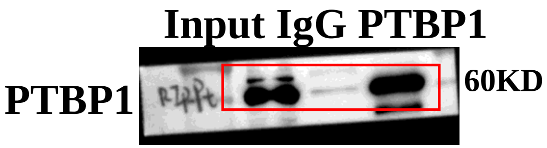

Figure4D-E

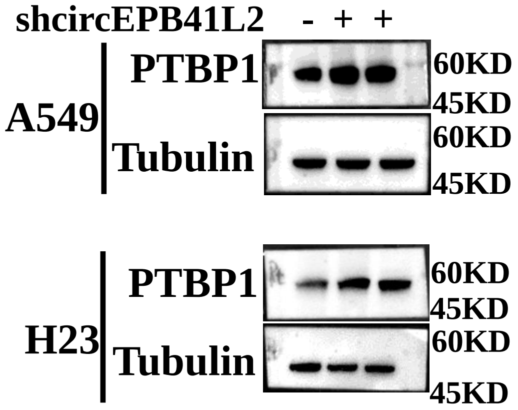

Figure4F-G

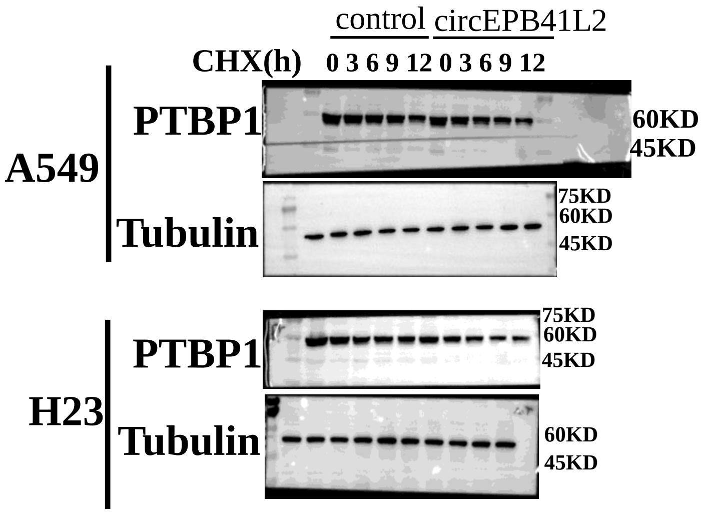

Figure4H-I

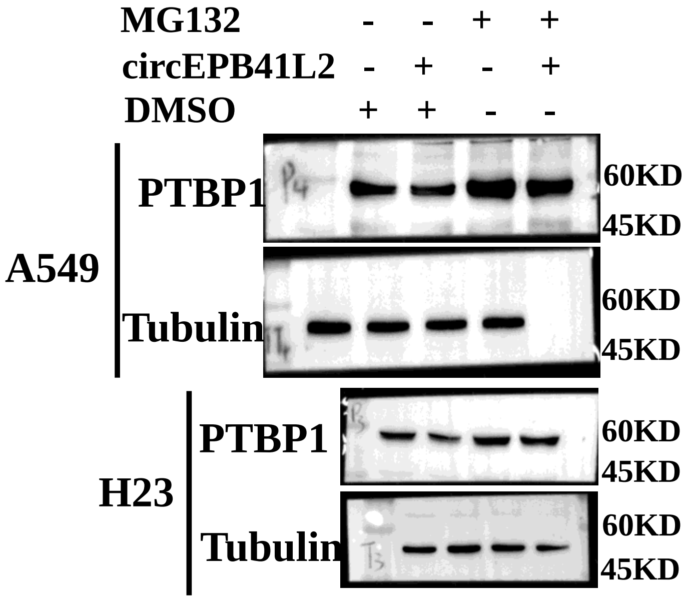

Figure4J

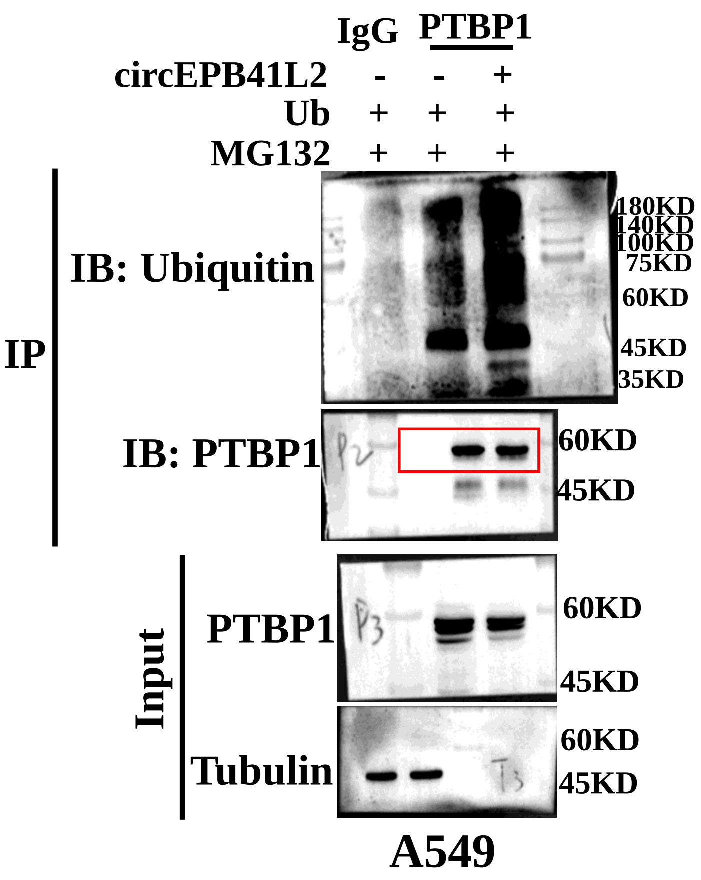

Figure4K

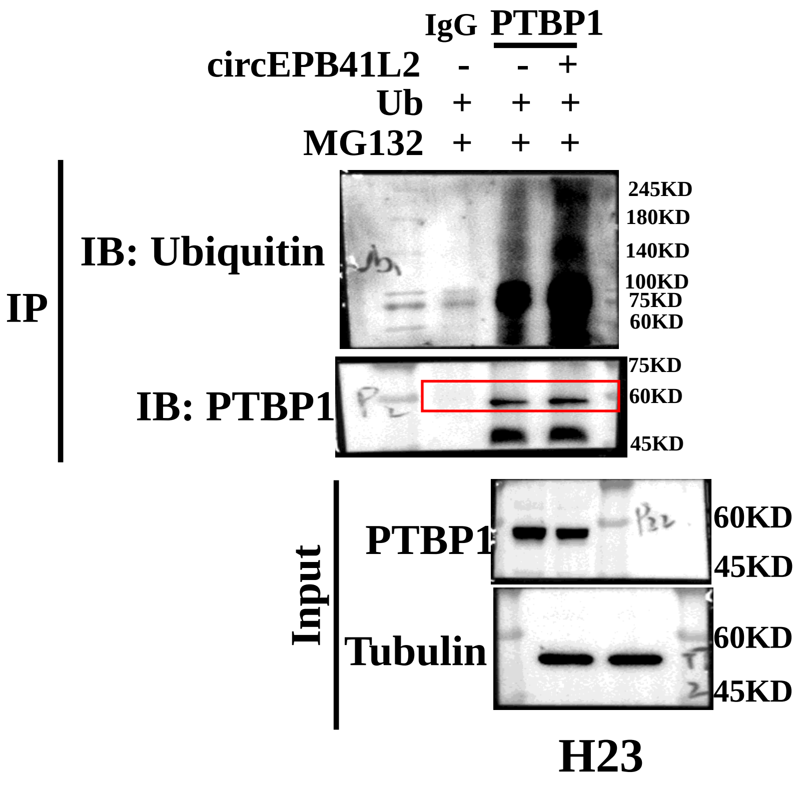

Figure4M

A549

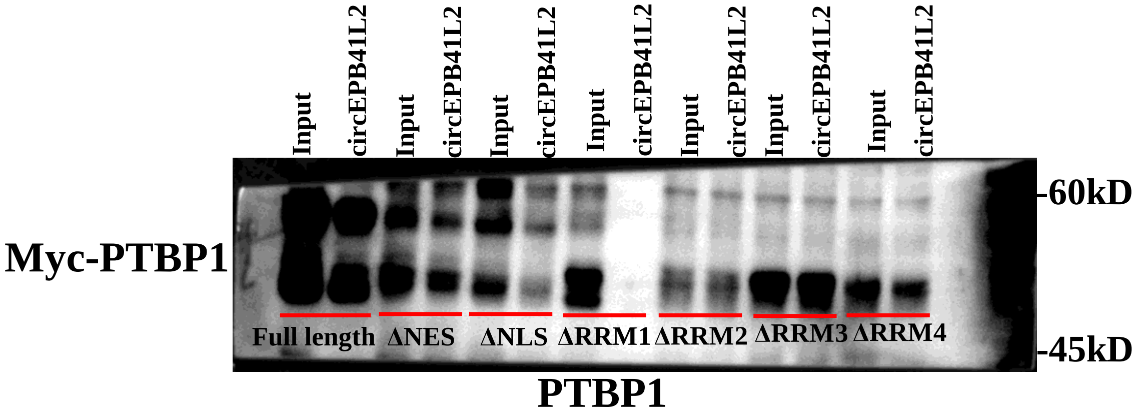

Figure4N

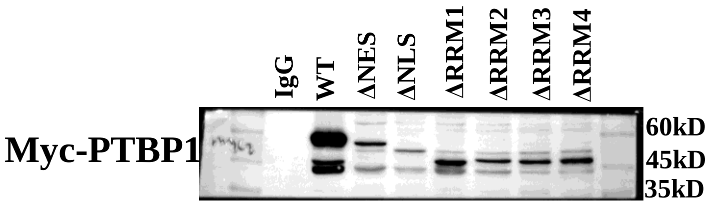

A549

Figure4O

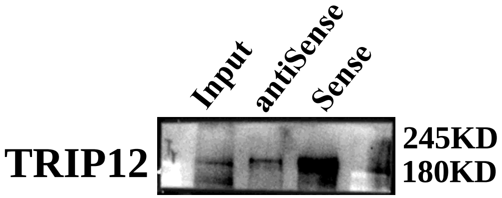

A549

Figure4P

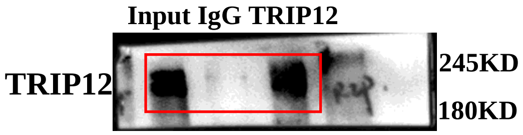

A549

Figure5B

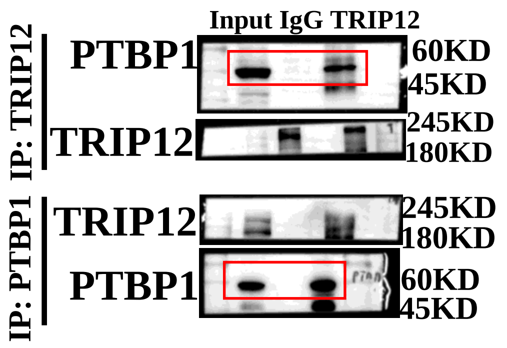

A549

Figure5C

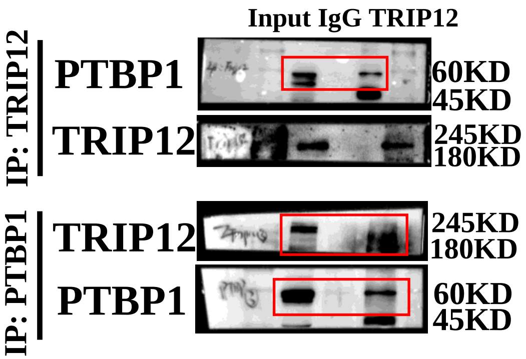

H23

Figure5F

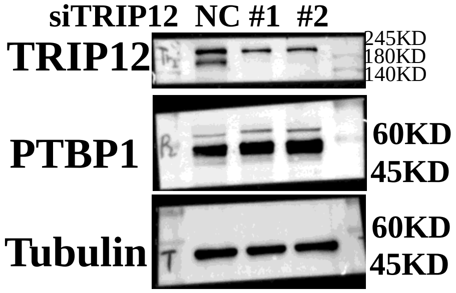

A549

Figure5G

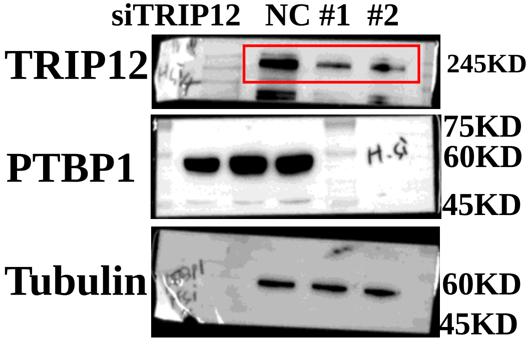

H23

Figure5H

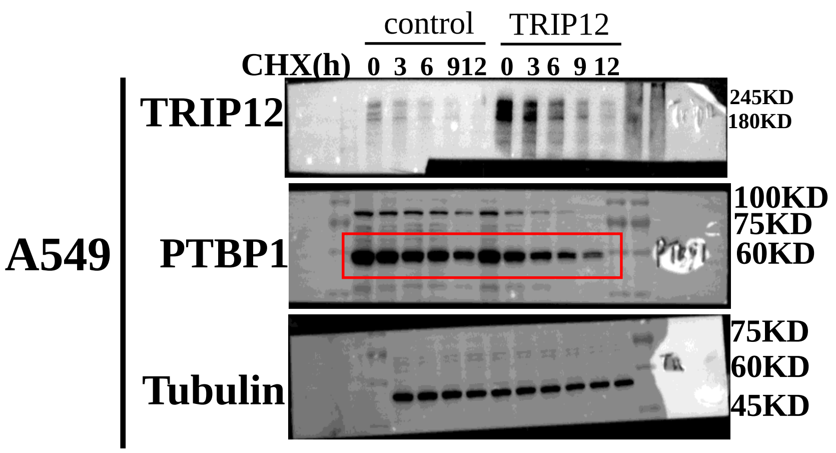

Figure5J

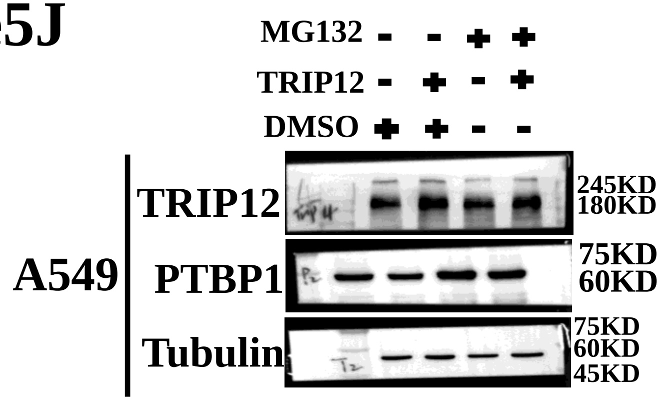

Figure5I

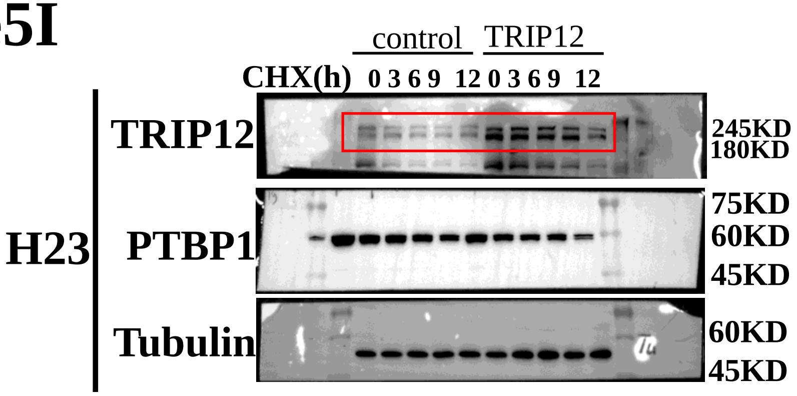

Figure5K

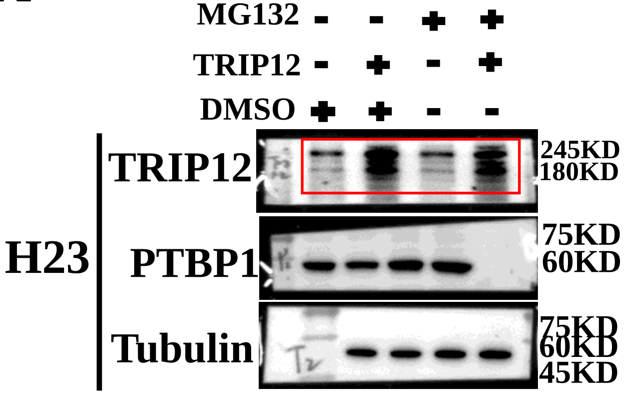

Figure5M

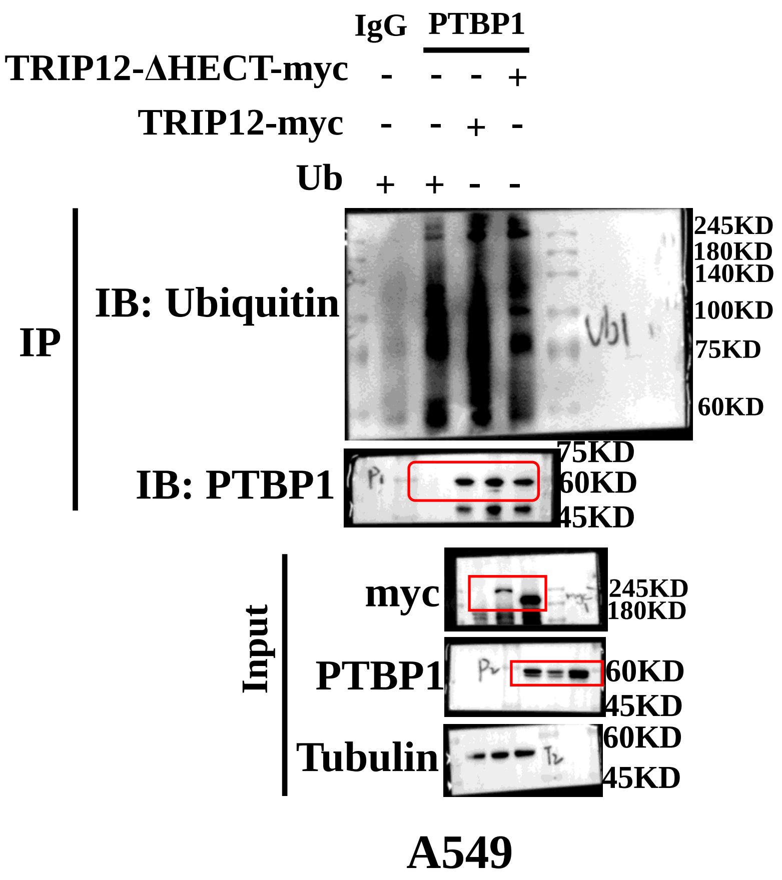

Figure5N

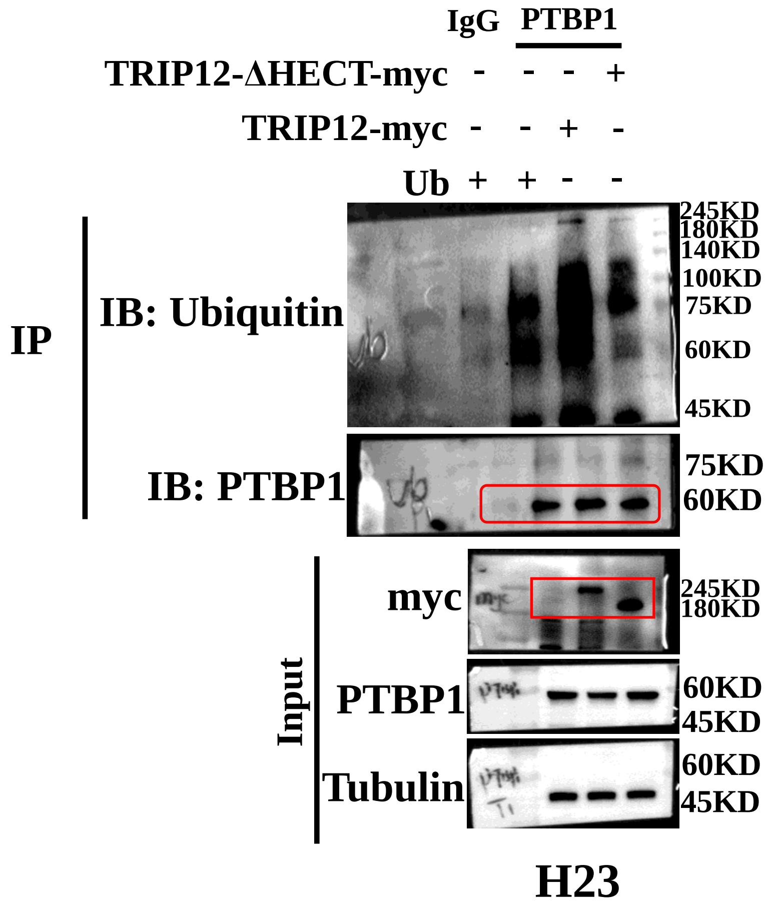

Figure 6A

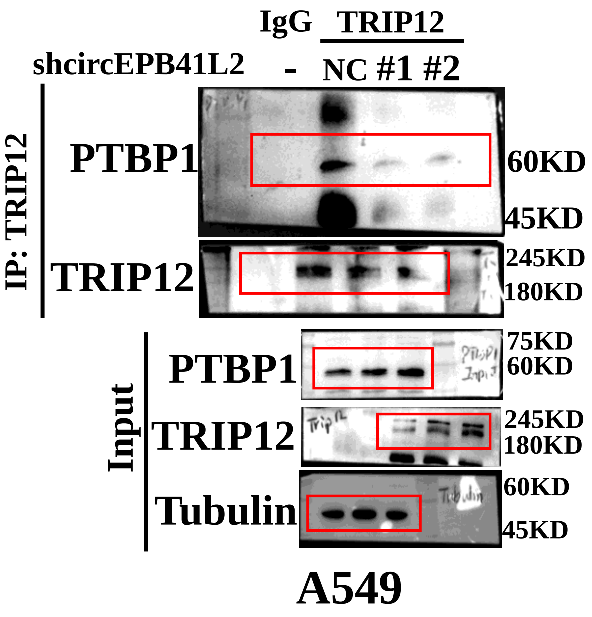

Figure 6B

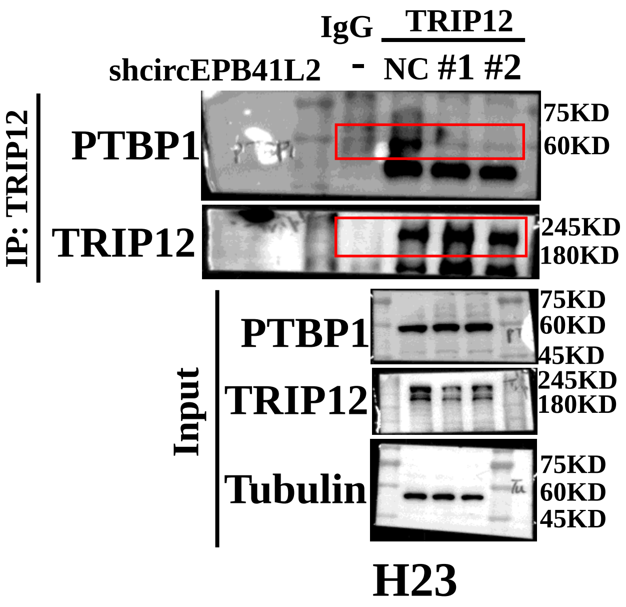

Figure 6C

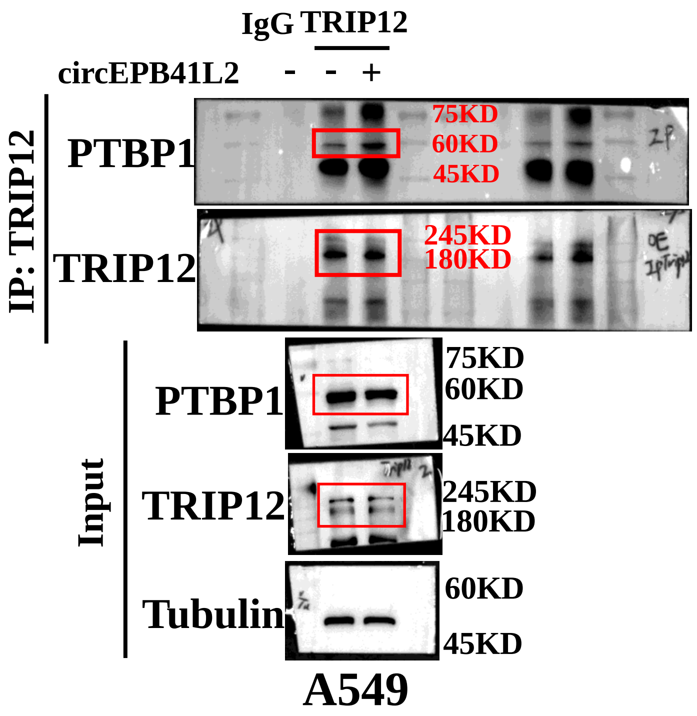

Figure 6D

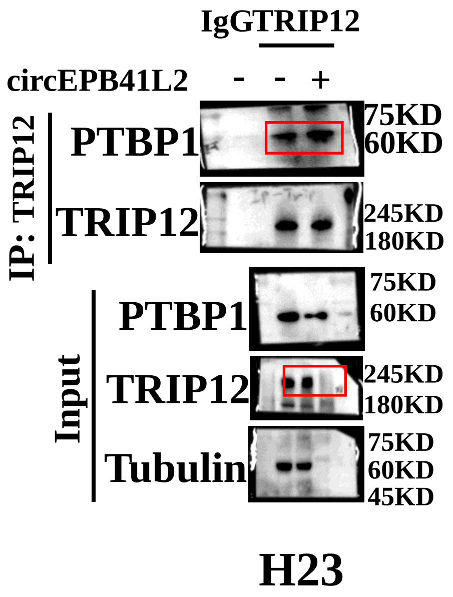

Figure 6E

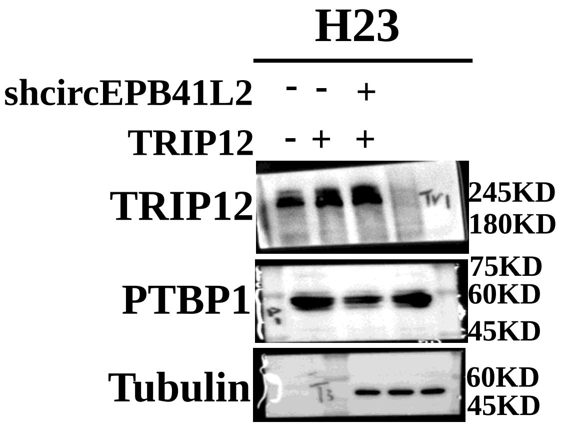

Figure 6F

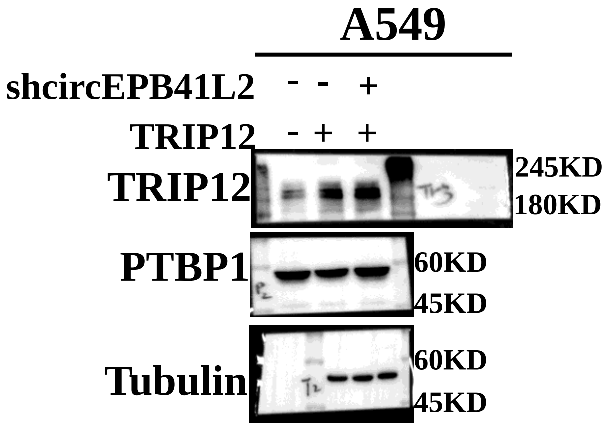

Figure 6G

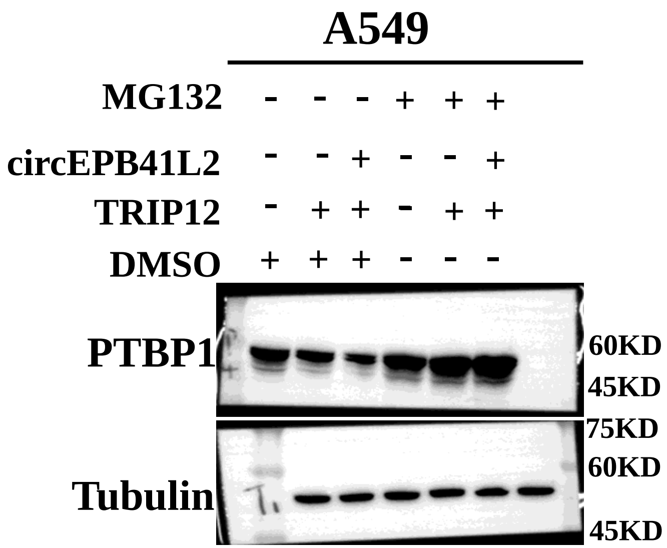

Figure 6H

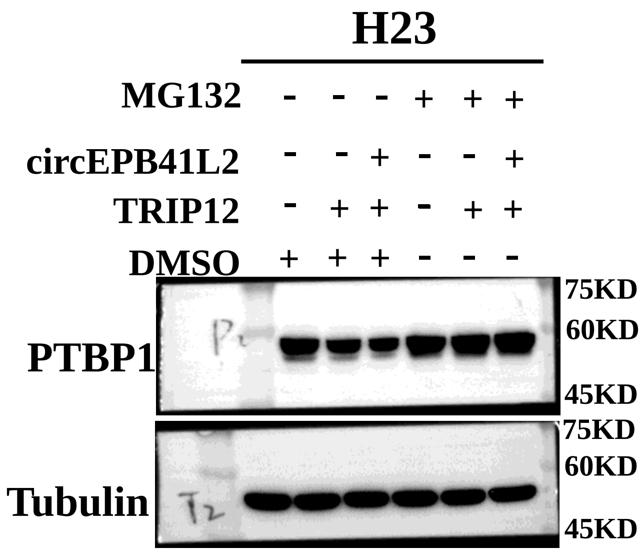

Figure 6I

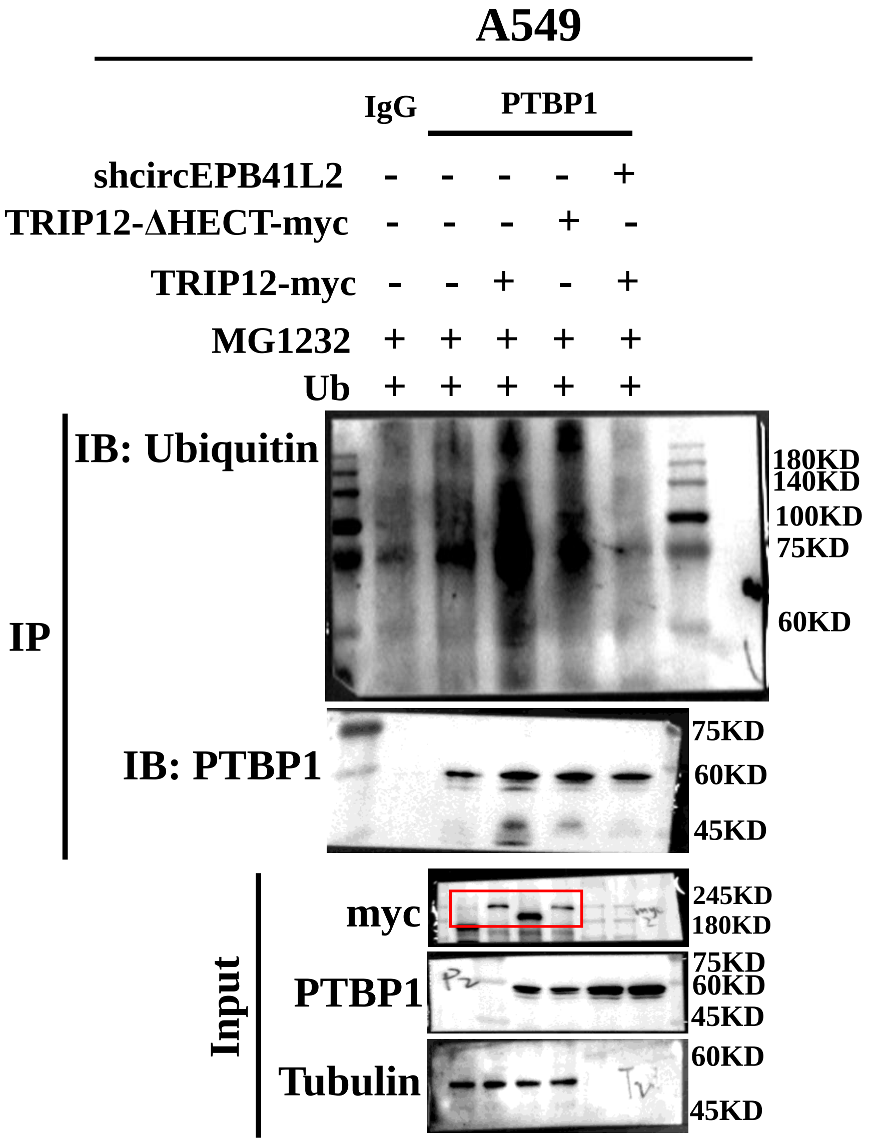

Figure 6J

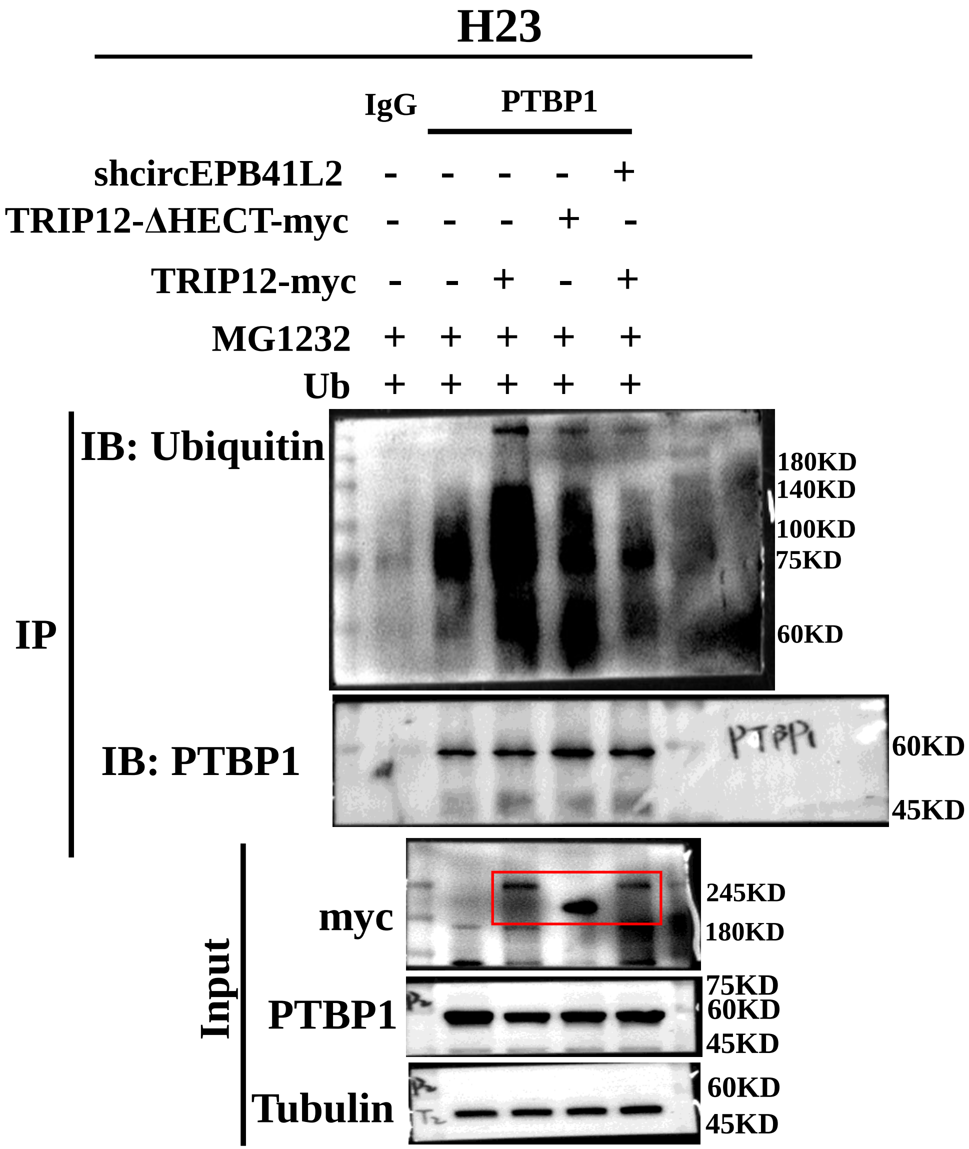

Figure 7B

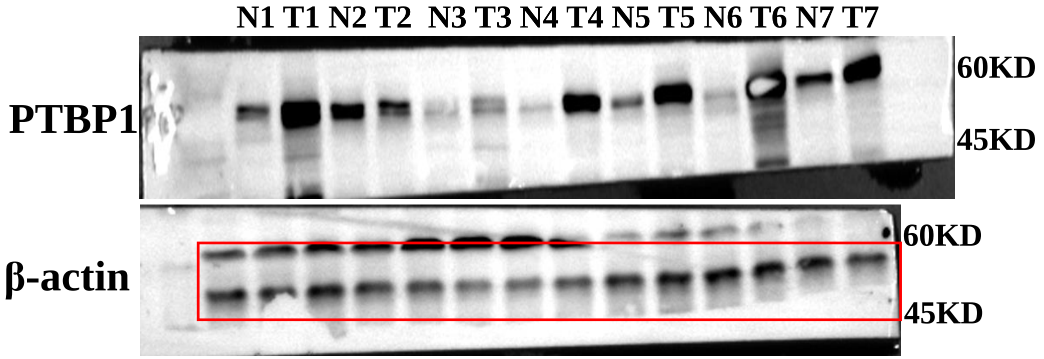

Figure 7G

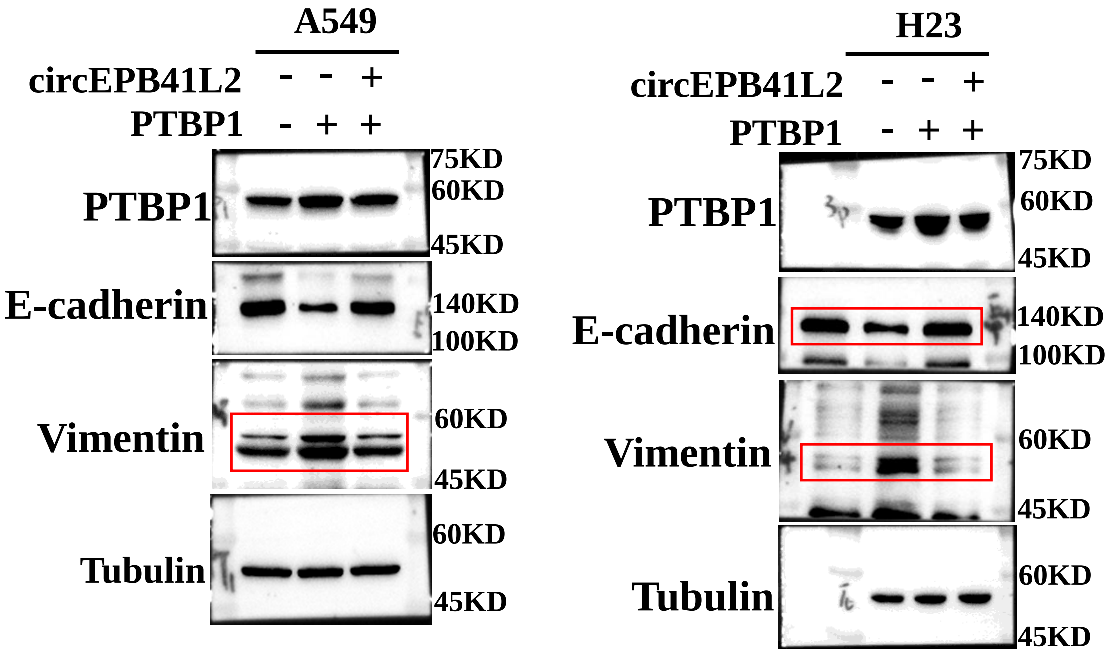

Figure 7H

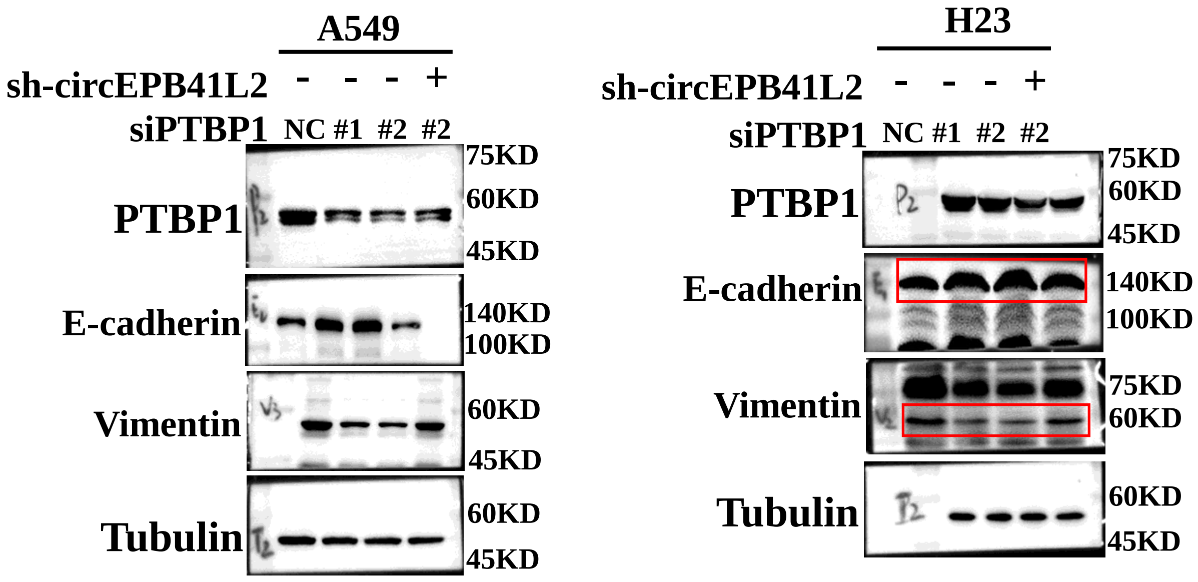

Figure8E

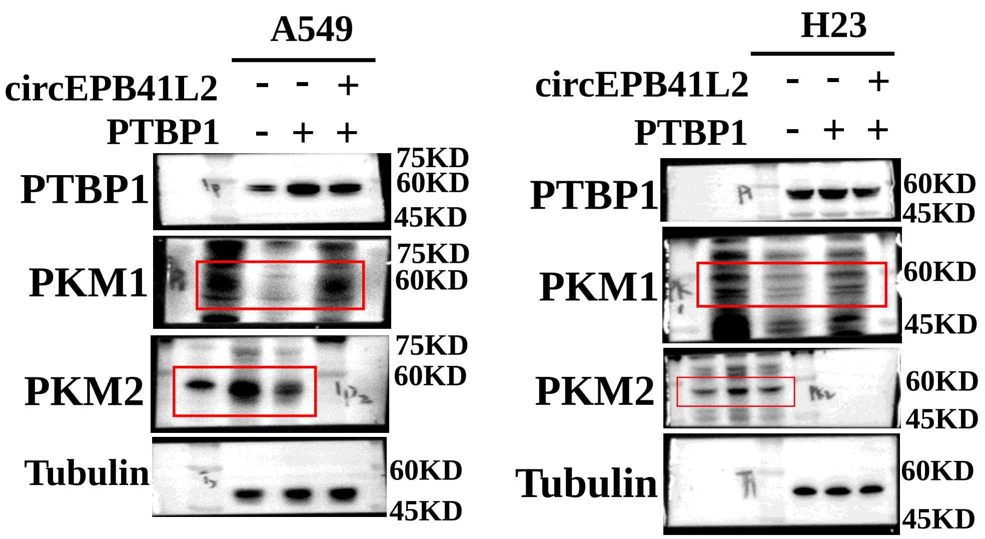

Figure8F

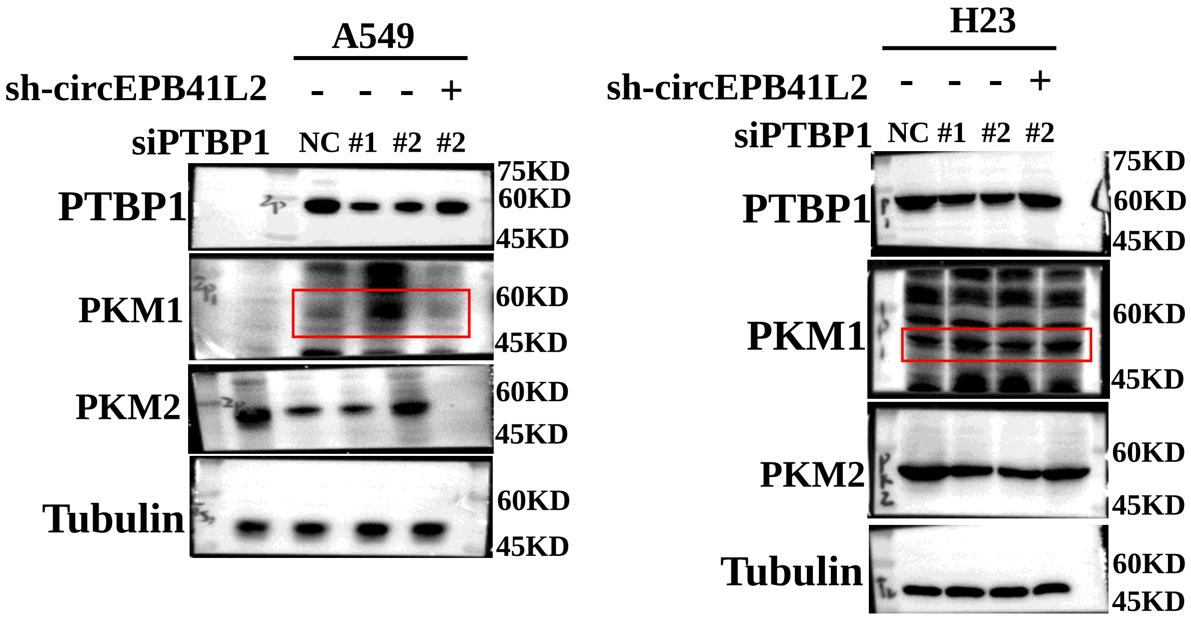

Supplement: Supplementary file 3 — Full and uncropped western blots [file 41420_2024_1836_MOESM3_ESM.pdf]
